# An ultra-fast reaction process for recycling lithium ion batteries *via* galvanic cell interaction†

**DOI:** 10.1039/d4sc06076h

**Published:** 2024-11-18

**Authors:** Long Ye, Zhilong Xu, Haiqiang Gong, Zhiming Xiao, Bao Zhang, Lei Ming, Xing Ou

**Affiliations:** a National Engineering Laboratory for High-Efficiency Recovery of Refractory Nonferrous Metals, School of Metallurgy and Environment, Central South University Changsha 410083 PR China ouxing@csu.edu.cn

## Abstract

The efficient realization of a closed-loop process is an ultimate goal for reusing spent lithium-ion batteries (LIBs), yet the complicated recycling processes of leaching and purification in an acid atmosphere are totally different compared with the regeneration method of the cathode precursor in alkali solution, inevitably resulting in the redundant consumption of acid/ammonia solutions and increased burden for a green environment. Herein, considering the advantages of selective extraction and similar chemical surroundings for recovery and regeneration, ammonia-leaching has been proposed to achieve short-process closed-loop recycling with effective impurity removal. Particularly, benefiting from the galvanic cell interaction, the sluggish reaction rate and relatively harsh reaction conditions of ammonia-leaching are well addressed. High leaching efficiency can be achieved within 10 min, where nearly 80% valuable metals are extracted in the initial 1 min. Notably, this leaching solution, after purification, can be used to directly synthesize the cathode precursor through the commercial alkali co-precipitation method. This process is superior to the acid leaching system, which requires the use of acid–base solutions back and forth to adjust pH for metal extraction and material regeneration. Compared to the traditional solid-to-liquid reaction with a shrinking core model, the solid-to-solid reaction with galvanic cell interaction substantially addresses the inherent issue of sluggish leaching efficiency, exhibiting much stronger competitiveness in the leaching rate and environment cost. Thus, it provides prospects to achieve large-scale recycling and regeneration of spent LIBs simultaneously in the whole-process alkali-atmosphere.

## Abstract 2

An ultra-fast, high-efficient and closed-loop ammonia-leaching process is proposed to provide a new perspective for spent LIB recycling.

## Graphical abstract

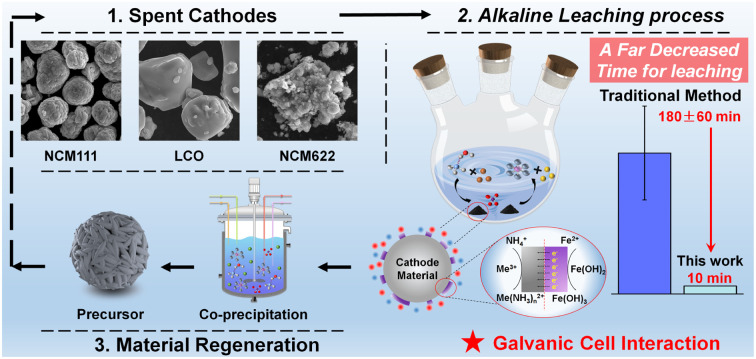

## Introduction

Due to economic development and technological progress, the recycling of valuable secondary resources is inevitable to maximize the utilization of valuable resources, especially strategic scarce metals.^1–4^ Spent lithium ion batteries (LIBs), called urban mines, contain a variety of valuable metals, like lithium (Li), nickel (Ni), cobalt (Co) and manganese (Mn). It is predicted that the growth of spent LIBs is entering an explosive phase, because of the fast spreading of electric vehicles in recent years.^5^ Among the current recycling methods, the hydrometallurgical process with its relative advantages, such as low energy consumption, high extraction efficiency and low environmental pollution, has the prospect of large-scale application in battery recycling. Still, difficulties in the optimization of the complicated impurity removal process and the seamless linkage within processes of leaching and regeneration need to be solved urgently.^6^

The ammonia-leaching process, as one of the conventional metal extraction techniques, is known for its characteristic selectivity, exhibiting a good adaptability to recover transition metals (Me = Ni, Co, and Mn) from the spent LIBs. After the leaching process, the targeted metals Li, Ni, Co, and Mn are mainly enriched in the leachate in the forms of isolated Li^+^ and [Me(NH_3_)_*n*_]^2+^ complexes, while the impurities are prone to be left in the leaching residue. Notably, the [Me(NH_3_)_*n*_]^2+^ complexes in the ammonia leachate share very similar compositions and chemical surroundings to the regenerated cathode precursor during the co-precipitation, providing a possibility to realize a closed-loop short-process of recycling and regeneration without the introduction of an enormous amount of acid solution.^7,8^ Unfortunately, the ammonia-leaching process exhibits intrinsically fatal flaws and a rather sluggish reaction rate and unfavorable side reactions which will result in an overlong reaction time and unsatisfactory leaching efficiency.^9–11^ Generally, the reaction time for the ammonia-leaching process always requires more than hours or even days, while the leaching efficiencies of Li, Ni, Co and Mn cannot all be greater than 95%. These unacceptable leaching results almost offset the advantages of the ammonia-leaching process in other aspects. Therefore, as long as the above problems can be solved properly, the ammonia-leaching can provide a strong application prospect.

According to previous research,^12–16^ the critical issue leading to the slow leaching kinetics of ammonia-leaching is the unfitness of the reduction process. The commonly soluble reducing agents during the leaching process include hydrogen peroxide (H_2_O_2_), sulfite (SO_3_^2−^), low-valence metal ions and some organic reagents with reducing properties. It is noted that these reducing agents well-applied in acid-leaching usually display unsatisfactory performance in alkaline surroundings, resulting in obvious defects hindering their application in ammonia-leaching.^17–19^ For instance, H_2_O_2_, as a weak acid, will rapidly decompose and release oxygen under alkaline conditions, while sulfite easily forms complex compounds with Mn-ions to wrap on the surface of unreacted particles, impeding further leaching of metals.^13,14,20^ Additionally, organic reagents exhibit reducing properties attributed to functional groups such as hydroxyl and aldehyde.^21^ However, their high cost and acidic nature pose challenges within alkaline systems, thereby restricting their potential for further development. Therefore, there is an urgent need to investigate a reducing agent that performs effectively in an alkaline-leaching system, remains in the leaching residue without compromising the purity of the leachate, and ultimately leads to the high recovery of valuable metals.

Herein, a delicate strategy involving galvanic cell interaction for rapid ammonia-leaching has been developed in this work. Specifically, Fe^2+^ ions are introduced to *in situ* form a solid phase of Fe(OH)_2_ nanograins with a homogeneous distribution, which creates galvanic cells upon contact with the spent NCM111 material. Benefiting from this mechanism, the electron transfer between NCM and Fe(OH)_2_ can greatly accelerate the reaction rate, intrinsically overcoming the sluggish reaction rate. Meanwhile, it is noted that the reaction product Fe(OH)_3_ is considered as an impurity and remains in the leaching residue, which has no effect on the subsequent leaching process. This is ascribed to the fact that the redox reaction is divided into two half reactions which occurred separately at their individual electrode, while most impurity metal hydroxides are insoluble and precipitated into the residue. Moreover, the optimized strategy also improves the leaching efficiency of valuable metals. Most of Li and Ni are extracted into the leachate, and 94.4% Co can be leached out, which can be completely recovered by the second round of leaching. In the further analysis, different cathode materials are adopted to prove the feasibility of the reaction mechanism proposed in this work. Both single-crystal LiCoO_2_ and spent NCM622 are recycled successfully with excellent leaching efficiency, which provides an advantage and efficiency for close-loop regeneration within the whole-process alkali-atmosphere.

## Experimental

### Materials

In order to get clear experimental results and reveal the underlying reaction mechanism, the exploratory research was mainly based on the analysis of the leaching behavior of main cathode materials NCM111 (LiNi_0.33_Co_0.33_Mn_0.33_O_2_). For further verification of its feasibility, the experimental subject was replaced with other materials to be recycled, including LCO (LiCoO_2_) and NCM622 (LiNi_0.6_Co_0.2_Mn_0.2_O_2_). All spent LIB cathode materials were purchased from Zhejiang Huayou Cobalt Co., Ltd. In addition to these experimental subjects, the other reagents involved were analytically pure. Notably, the alkaline solution for leaching was composed of NH_3_·H_2_O (25–28 wt%), (NH_4_)_2_SO_4_ (>99 wt%) and deionized water. As for the reducing agent, ferrous ion salt, in the form of FeSO_4_·7H_2_O (>99 wt%), played a crucial role in the leaching process.

### Leaching process

The alkaline solution was prepared with a certain amount of NH_3_·H_2_O, (NH_4_)_2_SO_4_ and deionized water, which were mixed together in a three-necked flask. Then, the temperature of the prepared solution was adjusted with a water bath and monitored by using a mercurial thermometer. When the specified temperature was reached and tended to be stable, the cathode powder to be leached was added into the solution and dispersed evenly by continuous magnetic stirring. After that, FeSO_4_·7H_2_O was added in the same way, which also represented the starting point of leaching. By recording the time, a small amount of the leachate was fetched out at 1, 2, 3, 4, 5 and 10 min for further element concentration detection to calculate the leaching rate of metals. The calculation formula was reported in our previous work. All of the leaching processes were terminated at 10 min, and thus the leaching residue could be separated and dried at 60 °C for further analysis. Moreover, based on the above leaching process, a detailed explanation of several factors and their influence on the leaching results was also provided, mainly including the usage amount of reagents and temperature.

### Analytical method

During the leaching process, the leachate and residue were the main object of analysis, which were preliminarily tested by ICP (inductive coupled plasma emission spectrometer, ICAP7400 Radial, USA) and XRD (X-ray diffraction, PANalytical/Empyrean 2, Netherlands), respectively. Furthermore, UV-vis (ultraviolet-visible spectroscopy) was conducted for the characteristic peak detection of substances in the leachate. SEM (scanning electron microscopy, JSM-7900F, Japan) was conducted for the morphology observation of the cathode material to be leached and the leaching residue, and EDS (energy dispersive spectroscopy) was adopted in the meantime to visually picture the element distribution and the ratio of the elements. In addition, the Pourbaix diagram, namely the Eh–pH diagram, was depicted to theoretically discuss the phase transition of each metal, thus providing a reliable prediction and explanation for the leaching process.

## Results and discussion

### Reaction mechanism for ammonia leaching

**Fig. 1:**
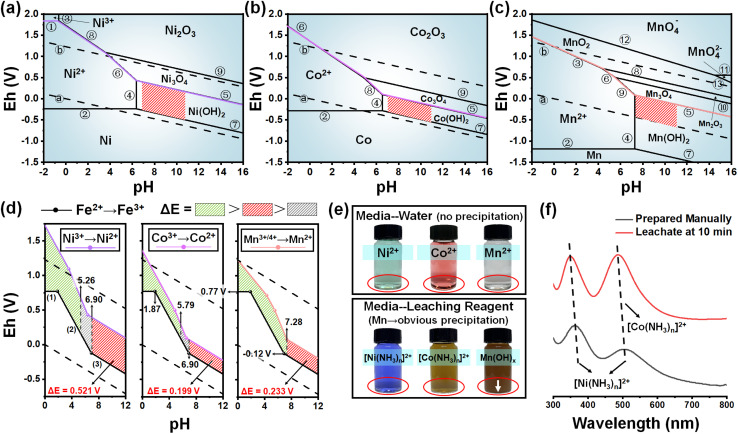
The Pourbaix diagram of the (a) Ni–H_2_O system, (b) Co–H_2_O system, and (c) Mn–H_2_O system. (d) The partial area comparison of the Fe–H_2_O system with Ni, Co, and Mn–H_2_O systems. (e) The real picture of the three metal ions in different solutions. (f) The UV-vis spectra of the leachate and control group.

The Pourbaix diagram of the Me–H_2_O system has always been introduced to explain the mechanism of the leaching process, especially for pH control and product prediction. Hence, various Me–H_2_O systems under standard conditions are provided, including Ni–H_2_O (Fig. 1a), Co–H_2_O (Fig. 1b), Mn–H_2_O (Fig. 1c) and Fe–H_2_O (Fig. S1†). The related thermodynamic data are collected from the chemical handbook and other reported research studies, which are provided in Tables S1–S4.†^22,23^ It is worth mentioning that the area enclosed by two dotted lines (a and b) represents the stable area in the water-based solution. In this way, the possible products should be located between these two dotted lines, which can be obtained by adjusting the pH of the solution. According to the literature,^23,24^ the stable areas of the metal ammine complex during the ammonia-leaching process are marked with red slashes in Fig. 1a–c, which are included in the area of low valent metal hydroxide. Owing to the high valence states of Ni, Co and Mn (+3 or +4) in the spent cathode, a reduction process is necessary before complexing with ammonia. As a result, Fe^2+^ exhibits excellent adaptability and can effectively function as a reducing agent within the ammonia leaching system.

By comparing the Pourbaix diagrams, the reduction mechanism of Fe^2+^ during the leaching process can be elucidated, as depicted in Fig. 1d. When the pH is between −2.00 and 1.87, the redox couple can be expressed as Fe^3+^/Fe^2+^ and its potential is maintained at 0.77 V. Subsequently, when the pH moves towards alkaline conditions, the oxidation state changes from Fe^3+^ to Fe(OH)_3_ because of quite a low solubility product constant of Fe(OH)_3_ (*K*_sp_ = 1.00 × 10^−38^ mol^4^ L^−4^), while the potential gradually decreases with the increased pH.^25^ As the pH value exceeds 6.90, the reduction state transforms from Fe^2+^ into Fe(OH)_2_ due to the still low solubility constant (*K*_sp_ = 4.87 × 10^−17^ mol^3^ L^−3^).^25^ Through the chemical equations (1)–(3) below, the corresponding formulae (4)–(7) for the changes in Eh with pH can be derived, which are drawn as black solid lines in Fig. 1d.

1 Fe^3+^ + e^−^ = Fe^2+^

2 Fe(OH)_3_ + 3H^+^ + e^−^ = Fe^2+^ + 3H_2_O

3 Fe(OH)_3_ + H^+^ + e^−^ = Fe(OH)_2_ + H_2_O

4 Δ*G* = Δ*G*^*θ*^ + *RT* ln *Q* = −*nFE*

5 *E*_(Fe^3+^/Fe^2+^)_ = −Δ*G*_1_^*θ*^/*F* − (2.303*RT*/*F*) × lg[*c*_(Fe^2+^)_/*c*_(Fe^3+^)_]

6 *E*_(Fe(OH)_3_/Fe^2+^)_ = −(2.303*RT*/*F*) × 3 × pH − Δ*G*_2_^*θ*^/*F* − (2.303*RT*/*F*) × lg[*c*_(Fe^2+^)_/*c*_(Fe(OH)_3__]

7 *E*_(Fe(OH)_3_/Fe(OH)_2_)_ = −(2.303*RT*/*F*) × pH − Δ*G*_3_^*θ*^/*F* − (2.303*RT*/*F*) × lg[*c*_(Fe(OH)_2_)_/*c*_(Fe(OH)_3__]

where Δ*G*, Δ*G*^*θ*^, *R*, *T*, *n*, and *F* represent the Gibbs free energy of the chemical reaction, the Gibbs free energy in the standard state, the ideal gas constant, the reaction temperature, the number of transferred electrons, and the Faraday constant, respectively.

Meanwhile, the colored solid lines for Ni^3+^ → Ni^2+^, Co^3+^ → Co^2+^ and Mn^3+/4+^ → Mn^2+^ can be extracted from Fig. 1a–c. Apparently, all colored solid lines are above the black solid line (Fig. 1d), indicating that the potential difference Δ*E* is always greater than 0, which is characteristic of thermodynamic feasibility.^26^ Therefore, no matter how the pH changes, the reducibility of Fe^2+^ or Fe(OH)_2_ can theoretically satisfy the reduction requirement of the valence state for Ni, Co and Mn from high +3/+4 to lower +2. Meanwhile, the pH of ammonia-leaching solution in this work is certainly alkaline, confirming the reducing agent function of Fe^2+^ in the form of chemical equation (3). It needs to be emphasized that, under alkaline conditions, the lines for metal reduction (Me^3+^ → Me^2+^) and the oxidation of Fe(OH)_2_ share the same slope of –(2.303*RT*/*F*), due to the same number of transferred electrons. Although the value changes as the reaction temperature changes, the calculated Δ*E* with these two parallel lines will always be greater than 0, representing that the reduction of high valence metal ions by introducing Fe^2+^ is theoretically practical.

The reduced metal ions (Me^2+^, including Ni^2+^, Co^2+^ and Mn^2+^) are prone to exist in the form of [Me(NH_3_)_*n*_]^2+^ instead of Me(OH)_2_, as the condition is controlled in the related stable area marked with red slashes in Fig. 1a–c. Practically, the color change of Me^2+^ in two different solutions of water and leaching reagent, as displayed in Fig. 1e, is solid evidence for the generation of [Me(NH_3_)_*n*_]^2+^ during the leaching process. The limpid solution with a deep blue color is [Ni(NH_3_)_*n*_]^2+^. The slightly cloudy solution with an earthy yellow color is [Co(NH_3_)_*n*_]^2+^. The obvious precipitate with a brown color is MnOOH, originating from Mn(OH)_2_ (white color) after oxidation. The stable area of Mn(OH)_2_ in Fig. 1c is intersected by line a, unlike Ni(OH)_2_ and Co(OH)_2_, representing that Mn(OH)_2_ in solution can exhibit both stability and instability due to its strong reducing ability. Hence, it is speculated that most of the Ni can be leached, and a small portion of Co and a large amount of Mn remained in the residue. This result is further proved with a series of experiments, and the optimizing strategy is also discussed. Additionally, the leachate at 10 min was further characterized by UV-vis spectroscopy, as illustrated in Fig. 1f and S2.†^27–29^ It has been confirmed that the leachate consists of mixed ammine complexes, and is similar to the manually prepared ammine solution. This indicates that the valuable metals are reduced and complexed into the leachate. It should be noted that the characteristic peak for [Mn(NH_3_)_*n*_]^2+^ is not discernible, falling within the wavelength range of 200 to 300 nm. As observed in Fig. S2(e1),† the presence of other peaks within the 200–300 nm range overlaps with the expected characteristic peaks of [Mn(NH_3_)_*n*_]^2+^, making them difficult to detect distinctly.

**Fig. 2:**
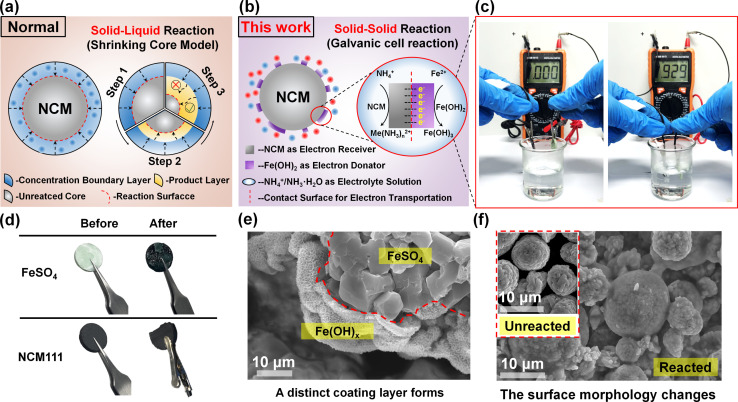
The schematic diagram of the (a) shrinking core model, and (b) galvanic cell interaction in this work. The real pictures of the (c) external circuit current detection test, and (d) the prepared electrodes before and after the test. The SEM images of the (e) FeSO_4_ electrode and (f) NCM111 electrode after the test.

Through the thermodynamic feasibility analysis and leachate characterization, the reduction effect exhibited by galvanic cell interaction still needs to be confirmed. In order to further unveil the ultra-fast reaction rate of ammonia-leaching, the kinetic explanation is necessary. For better understanding, the common reduction process and galvanic cell interaction during alkaline leaching are schematically expressed and compared.^30^ As demonstrated in Fig. 2a, the general leaching process mainly uses a soluble reducing agent to trigger the liquid–solid reaction, so that it can be summarized in the shrinkage nuclear reaction model. Such a leaching process is very rapid under acid leaching conditions, but there is a major problem under alkaline leaching conditions. Most of the soluble reducing agents are hardly used under alkaline conditions, which will easily lead to the formation of complex precipitate which adheres to the outer layer of unreacted materials during the alkaline leaching process.^31^ This hinders the diffusion of leaching/reducing agents and greatly prolongs the required time for the subsequent chemical reaction. In order to avoid the above phenomenon, the ferrite salt used in this work will *in situ* generate ferrous hydroxide under alkaline conditions, which can create numerous tiny galvanic cells based on the principle of solid–solid reaction rather than solid–liquid reaction, as displayed in Fig. 2b. Under this circumstance, the spontaneous electron transfer will greatly speed up the reaction rate after the formation of a galvanic cell, which is much higher than that of ordinary redox processes. This finding of galvanic cell interaction is strongly confirmed by connecting the external circuit of pressed NCM111 and FeSO_4_ electrodes. As shown in Fig. 2c, after solution immersion, it exhibits an obvious current of 0.929 mA, illustrating the process of electrochemical reaction.

More importantly, during this electrochemical reduction process, the oxidation and reduction reactions occur at their respective electrodes, rather than at the contact site.^32,33^ Due to this feature, it is difficult to form product layer wrapping on the material surface, guaranteeing the unhindered diffusion process of various reagents. This result can also be proved by the morphological changes in the NCM electrode after the test. The test lasted for 30 seconds, during which the surface of the FeSO_4_ electrode changed noticeably, and a portion of the NCM111 electrode gradually powdered into the solution, as displayed in Fig. 2d. At the mesoscopic level, a uniform layer is coated on the surface of the FeSO_4_ electrode (Fig. 2e). The outer layer is ascribed to a mixture of Fe(OH)_2_ and Fe(OH)_3_, according to chemical equation (3). Meanwhile, the powdered NCM111 collected after filtration also exhibits a visible change in surface and particle size, as depicted in Fig. 2f and S3,† further indicating that the corresponding half-reaction also occurs on the NCM111 electrode.

### The ultra-fast leaching process

The leaching process is intrinsically a redox reaction, the result of which is mostly influenced by the concentration of the reactant and temperature. In order to practically verify the reaction mechanism of galvanic cell interaction during the ammonia-leaching process, various reagents combined with corresponding conditions were investigated, while the leaching efficiency changes of each metal are summarized. In particular, the leaching efficiencies corresponding to the initial 1 min and final 10 min are indicated by the blue and red curves, respectively, so as to distinctively illustrate the influence trend of each factor.

**Fig. 3:**
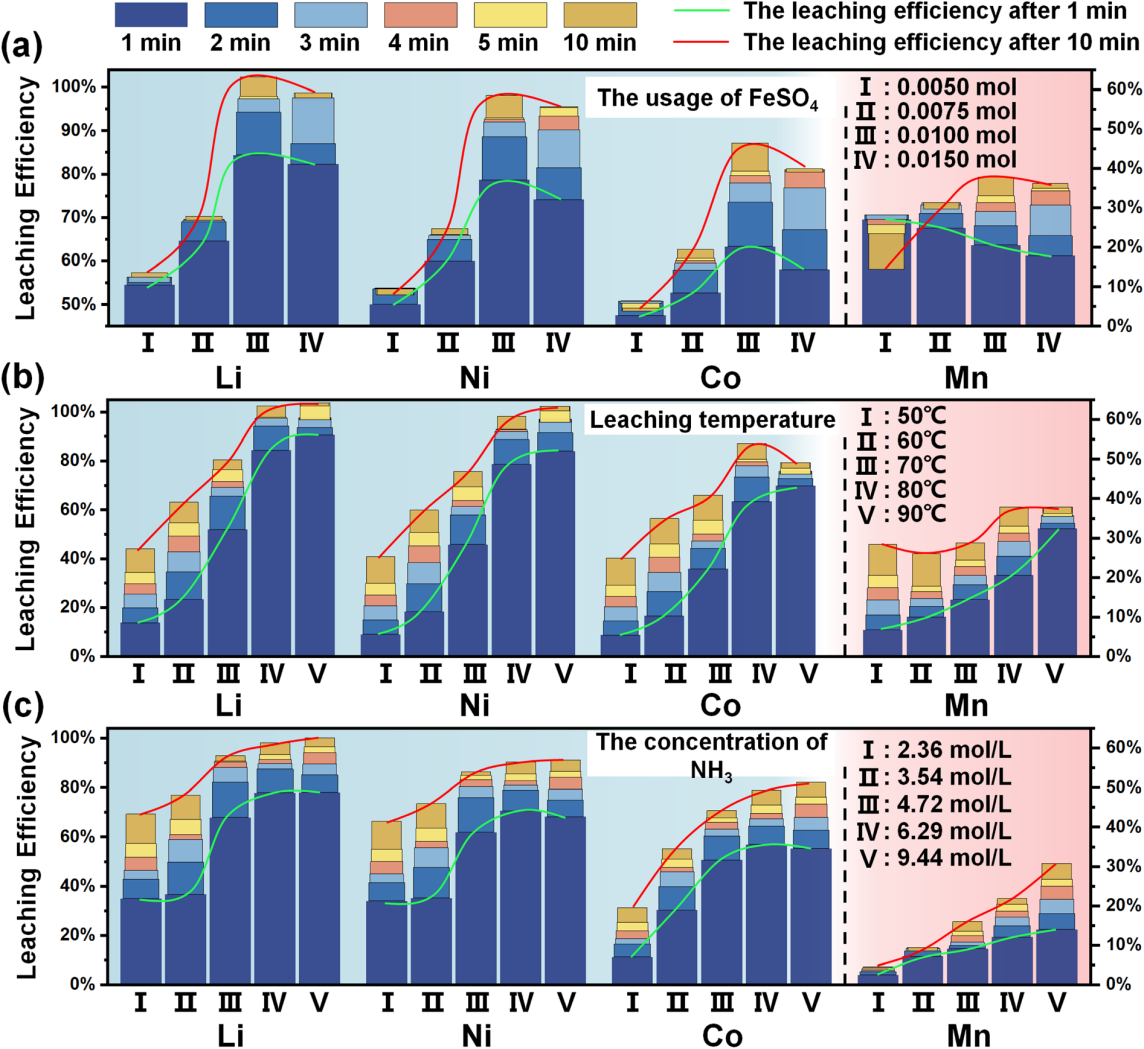
The leaching efficiency of valuable metals changes with the gradient of (a) FeSO_4_ usage, (b) leaching temperature, and (c) the concentration of NH_3_.

The leaching efficiency influenced by the usage amount of FeSO_4_ (Fe^2+^) is illustrated in Fig. 3a. An increase in the usage amount of FeSO_4_ corresponds to an enhanced production of Fe(OH)_2_, which in turn leads to the formation of a greater number of galvanic cells in conjunction with NCM111, thereby accelerating the leaching efficiency of metals. It is seen that the leaching efficiencies of Li, Ni, and Co increase with the augmented amount of FeSO_4_, while the maximum value is obtained when the usage amount of Fe^2+^ is 0.01 mol. However, when the addition amount gets continuously increased, the leaching efficiency starts to decline, especially for Co. This is attributed to the selective adsorption behavior of newly formed Fe(OH)_3_, originating from its carried negative charge endowed by the electrical double layer.^34,35^ Additionally, the leaching behavior of Mn is apparently different from that of Li, Ni and Co. Due to the instability of Mn(OH)_2_, both [Mn(NH_3_)_*n*_]^2+^ (which stayed in the leachate) and MnOOH (which precipitated into the residue) will form during the leaching process, while the latter directly affects the leaching efficiency of Mn. Certainly, slightly excessive usage of Fe^2+^ is beneficial to increase the ratio of [Mn(NH_3_)_*n*_]^2+^ by inhibiting the subsequent oxidation, while deficient usage results in phase transformation towards MnOOH. When the usage amount of Fe is 0.005 mol, the decreasing leaching efficiency over leaching time powerfully supports that Mn is extracted first and then oxidized subsequently. This problem is visibly alleviated by increasing the usage amount of Fe^2+^. Moreover, the change in the leaching residues at 10 min is complementary to the analysis of the leachate, as revealed by XRD patterns (Fig. S4†) and SEM images (Fig. S5†). The residues mostly consist of Fe_3_O_4_ and Mn_3_O_4_, while NCM111 gradually disappears, which coincides with the analysis above. Additionally, the leaching process without FeSO_4_ is discussed in Fig. S6,† underscoring the essential role and indispensability of FeSO_4_. The extraction of metals like Ni, Co and Mn is negligible, whereas the leaching rate of Li reaches around 6%. The constant valence state of Li, coupled with the constrained diffusion dynamics at the solid–liquid interface, may account for the extraction observed for Li. It is confirmed that ammonia solution cannot directly react with the cathode material without the presence of a reduction process.

Variations in temperature directly influence the rate of mass transfer, a critical factor in the electrochemical process for the migration of reactants and products. For Li and Ni as presented in Fig. 3b, their leaching efficiencies share a similar increasing trend to increasing temperature, exhibiting a positive correlation. For Co, the adsorption ability of Fe(OH)_3_ is also enhanced with the elevated temperature, causing a slightly decline in its leaching efficiency when it exceeds 80 °C. For Mn, such a wide range of temperature changes has little influence on its final leaching efficiency, though the leaching efficiency at 1 min is increased with temperature changes. The leaching efficiencies of valuable metals are given priority as indicators, leading to a conclusion that the optimal reaction temperature is 80 °C. Besides, the leaching residues exhibit an obvious gradual transformation process from NCM111 into Fe_3_O_4_/Mn_3_O_4_, as depicted in Fig. S7,† which is also confirmed by their morphological characteristics (Fig. S8†).

The introduction of NH_3_ exhibits two main functions, which help in building an alkaline environment and provide ligand molecule NH_3_ for a complex reaction, reflected by the pH and total NH_3_ concentration. The incorporation of NH_3_·H_2_O is primarily intended to ensure the maintenance of an optimal concentration of NH_3_, while the judicious addition of a modest quantity of (NH_4_)_2_SO_4_ serves as an effective means of pH modulation, as presented in Fig. S9.† To confirm the roles of NH_3_·H_2_O and (NH_4_)_2_SO_4_ in the leaching process, a series of experiments were carried out with a focus on their usage amount. The experimental data reveal that modulating the usage amount of NH_3_·H_2_O (Fig. S10a†) or (NH_4_)_2_SO_4_ (Fig. S10b†) independently results in a comparatively modest influence when contrasted with alterations in the usage amount of FeSO_4_ or variations in temperature. It is supported that the subtle variations in pH determines the feasibility of the reaction, while a major change in the total NH_3_ concentration is responsible for the leaching results. As confirmed, the combination of 120 mL NH_3_·H_2_O and 0.1 mol (NH_4_)_2_SO_4_ exhibits the best leaching efficiency. The analysis of the residues provides a consistent result, as presented in Fig. S11–S14.† Furthermore, by simultaneously changing NH_3_·H_2_O and (NH_4_)_2_SO_4_ in equal proportions, its pH value can be kept constant, as listed in Fig. S9c.† As depicted in Fig. 3c, the decreased total concentration has a noteworthy influence on the leaching efficiency, especially for Co and Mn. Thus, the leaching behavior of metals is basically revealed, and the stability of their reduction state (Co^2+^ and Mn^2+^) is inferior to that of Li^+^ and Ni^2+^ under the same conditions. A higher concentration of NH_3_ is required to form more stable ammonia complexes, otherwise a large proportion of metals will be lost into the residue.^34,35^ Additionally, XRD and SEM are further conducted for all residues to provide complementary evidence, as presented in Fig. S15 and S16,† respectively. Based on the above leaching results, the relative impact of these variables can be delineated as follows: the usage of FeSO_4_ > temperature > the total concentration of NH_3_.

**Fig. 4:**
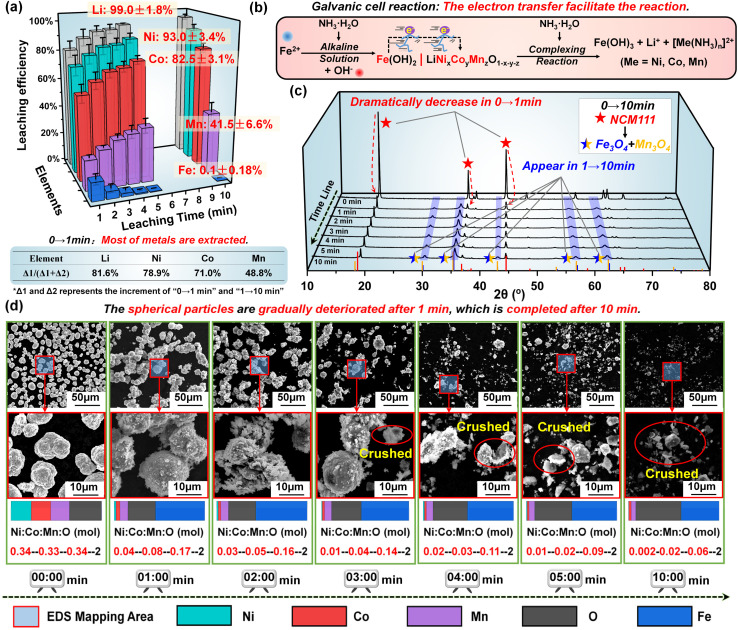
(a) The leaching efficiency of Li, Ni, Co, Mn and Fe with the increased time from 1 min to 10 min. (b) The expression of the leaching process. The changes in (c) the XRD pattern and (d) multiple enlarged SEM images for the leaching residues with the increased time from 1 min to 10 min.

Based on the above analysis, five repeated experiments were conducted under the optimal conditions to further demonstrate this ultra-fast alkaline leaching process. As illustrated in Fig. 4a, the three-dimensional bar graph visually confirms the ultra-fast rate of metal dissolution. The leaching process is basically completed in 10 min, and the leaching efficiency can reach 99 ± 1.8%, 93 ± 3.4%, 82 ± 3.1%, 42 ± 6.6% and 0.1 ± 0.18% for Li, Ni, Co, Mn and Fe, respectively. More importantly, in proportion to the final leaching efficiencies, 81.6% Li, 78.9% Ni, 71.0% Co and 48.8% Mn are extracted in the first 1 min, firmly substantiating that an ultra-fast reaction occurs during the leaching process. For error analysis, the standard deviation (SD) is adopted to analyze the data from five independent experiments, as listed in Table S5.† The decreased degree of deviation over time proves that the reaction is intense and fast initially and gradually levels off. Still, a small amount of Ni and part of Co is stuck in the leaching residue, which may be attributed to the adsorption of Fe(OH)_3_. A multi-step leaching process is adopted to address this problem, which will be discussed in the following adaptive research part. Generally, this leaching result in such a short leaching time can be a powerful advantage for ammonia leaching, which is entirely attributed to the unique reaction mechanism of galvanic cell interaction, as presented in Fig. 4b. Specifically, the introduction of Fe^2+^ in an alkaline environment can form Fe(OH)_2_ nanoparticles with a homogeneous dispersion. Along with continuous stirring, two solid phases, Fe(OH)_2_ and NCM111, will intimately come into contact and then constitute the galvanic cells. Benefiting from the participation of rapid electron transfer, this redox process is more accelerated instead of the traditional reduction reaction with limited kinetics, which explains the high leaching efficiency within such a short time. Moreover, due to the alkaline environment, Fe^2+^ ions end up in the residue without affecting the purity of the leachate.

In addition to the leachate, the changes in leaching residue with increased time from 1 min to 10 min were further investigated by XRD and SEM tests. As demonstrated in Fig. 4c, the intensities of characteristic peaks of NCM111 have dramatically decreased in the first 1 min, while the peaks representing Fe_3_O_4_ and Mn_3_O_4_ phases gradually appear (due to the dehydration of Fe(OH)_*x*_ and Mn(OH)_*x*_, in which *x* = 2, 3). Along with the increased leaching time, such changes are continued albeit less pronounced. The SEM images of leaching residues vividly illustrate the morphology changes over time, as displayed in Fig. 4d. On the whole, the spherical particles are gradually deteriorated after 1 min, which is completed after 10 min. It should be noted that the morphology changes lag behind the leaching outcomes, which is the result of the adherence of Fe(OH)_2_. Due to the solid-to-solid nature of the reaction process, island-like precipitates form *in situ* on the surface of NCM111, as evidenced by SEM images in Fig. 4d. As the reaction proceeds, the metal is leached into the solution, causing the NCM111 particles to diminish in size, while the island-like adherents grow gradually, temporarily maintaining the relative integrity of the particle structure. After three minutes, with a greater amount of metal leached out, the particle structure can no longer sustain stability, leading to disintegration and the appearance of numerous shell-like fragments with a spherical morphology. Based on the corresponding EDS tests (Fig. S17†), the element distribution and proportion strongly support these results. With the content of O as the benchmark for comparison, it can be found that the proportion of Ni : Co : Mn : O is changed from 0.34 : 0.33 : 0.34 : 2 (pristine NCM111) to 0.04 : 0.08 : 0.17 : 2 within 1 min. This result indicates that most of Ni, Co and Mn are extracted, which is consistent with the leachate analysis (Fig. 4a). More importantly, this also proves that there is no harmful behavior of the solid product layer hindering the dissolution of metals during the entire leaching process. It should be noted that, during the common ammonia leaching process, the solid product could intimately surround the unreacted spent NCM materials. In our work, due to the characteristics of galvanic cells, the electrode reactions occur separately and the reaction products are generated at their respective electrode, resulting in an accelerated kinetic rate and more complete leaching reaction.

### The adaptive analysis of the method

**Fig. 5:**
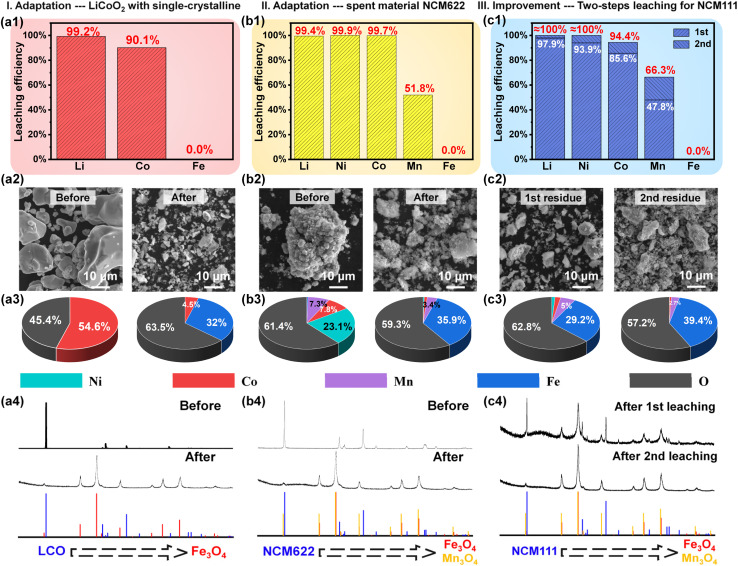
The analysis of recycling (a1–a4) the single-crystalline material LiCoO_2_, (b1–b4) the spent cathode material NCM622, and (c1–c4) the two-step leaching process.

In order to evaluate the adaptation of this method, more experiments on different spent cathode materials have been conducted under the same conditions. Firstly, it is generally acknowledged that materials with a single-crystalline structure have trouble dissolving during the leaching process, due to their larger micron-sized particles.^36^ Particularly, the presence of a solid by-product layer prevents the direct contact between the unreacted core bulk and the reaction reagents, unavoidably resulting in incomplete reactions. Whereas, as a classical member of single-crystal cathodes, LiCoO_2_ (LCO) is likely to be mixed with NCM materials during the recycling process, which meets the verification requirements and achieves a satisfactory effect in this work. As illustrated in Fig. 5a1, it confirms the adaptability of this strategy for single-crystal cathodes, where the leaching efficiencies of Li, Co and Fe reach 99.2%, 90.1% and 0% within 10 min, respectively. As displayed in Fig. 5a2, the large particles with a size of 5–20 μm are completely broken into smaller particles, as complementary evidence to confirm the adaptability of our process, which is further proved by the EDS test (Fig. S18†). The obtained element proportions of Co, O and Fe (Fig. 5a3) are in line with the above analysis. The XRD patterns indicate that the characteristic peaks of LCO disappear and are replaced by those of Fe_3_O_4_, as presented in Fig. 5a4. It is obvious that although the spent materials exhibit a more stable structure and larger size, they can still be well disposed and dissolved, due to the superiority of galvanic cell interaction. Moreover, the adaptability to various spent materials with different metal ratios was further verified by using waste LiNi_0.6_Co_0.2_Mn_0.2_O_2_ (NCM622). The leaching results in Fig. 5b1 show a satisfactory leaching efficiency of near 100% for Li, Ni and Co, simultaneously. Compared to LCO, the lower concentration of Co in NCM622 is advantageous for complete leaching, as it reduces the adsorption of Fe(OH)_3_ and prevents unnecessary agglomeration. Owing to the dynamic equilibrium that characterizes metal complexes, the concentration of free Co^2+^ ions incrementally increases in response to an increase in the total cobalt ion concentration, provided that the ammonia concentration is maintained constant. Then, the elevated free Co^2+^ ions will be partially adsorbed by Fe(OH)_3_. As a result, NCM622, which possesses the lowest cobalt content among LCO and NCM111, is less prone to adsorption by Fe(OH)_3_. Similarly, the reacted morphology (Fig. 5b2), the element distribution (Fig. S19†), the corresponding element ratio (Fig. 5b3) and XRD patterns (Fig. 5b4) of the leaching residue coincide well with the above leachate analysis.

Additionally, a two-step leaching process is introduced as an effective approach to further enhance the leaching efficiency and solve the loss of valuable metals, especially for Co. As displayed in Fig. 5c1, the valuable metals retained in the first-round leaching residue are successfully extracted after the second-round leaching process. Different from Li and Ni, it is inferred that there is a distribution ratio between the leachate and residue for Co and Mn during the leaching process, since the leaching efficiencies of the two steps are close in proportion. According to the leaching result in Fig. 3c, this distribution ratio is directly influenced by the concentration of NH_3_, pointing out a direction for the subsequent optimization. Moreover, according to the reacted particle morphology element distribution and phase structure (Fig. 5c2–c4 and S20†), the complete transformation from spent NCM111 into Fe_3_O_4_ is further confirmed, demonstrating the overwhelming advantages of ammonia leaching.

As anticipated, the designed strategy demonstrates excellent adaptability in handling a variety of spent cathodes with larger particle size or different metal compositions. The leaching behavior of each metal is further confirmed, offering guidance on reducing metal loss to the residue. For instance, Co and Mn in the residue can be retransferred into the leachate *via* a secondary leaching process, which mainly relies on the distribution ratio of these metals between the leachate and residue. It is important to note that, no matter the presence of intrinsic impurities or additional reaction incorporation, the Fe element consistently ends up in the residue across all cases without compromising the purity of the leachate.

### The evaluation of the whole process

To develop a sustainable and closed-loop recycling process is a crucial approach for achieving the efficient utilization of valuable secondary resources.^37^ Spent LIBs, being a high-value recyclable secondary resource, exhibit complexities in terms of type, elemental composition, and material structure.^38–40^ These characteristics inevitably impose greater demands on the recycling process, particularly with regard to adaptability and efficiency.

**Fig. 6:**
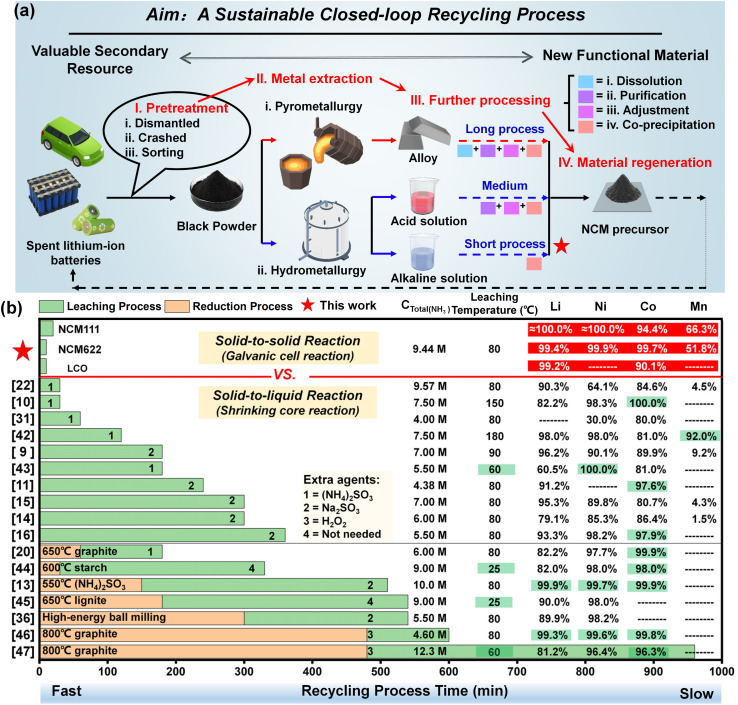
The comparison of (a) the entire process with other recycling methods, (b) various technical indices with other reported literature related to ammonia leaching systems.

As depicted in Fig. 6a, a recycling process for spent LIBs can typically be summarized into four steps: pretreatment, metal extraction, further processing, and material regeneration. Apparently, the metal extraction step is the most challenging in achieving efficient recycling without sacrificing adaptability. The adoption of a pyrometallurgical process for the treatment of spent cathodes (black powder) to achieve the regeneration material involves a lengthy procedure, leading to a significantly low metal utilization efficiency. Additionally, the process is characterized by high energy consumption and is accompanied by the generation of difficult-to-manage gas pollutants, which collectively results in its limited practical applicability.^38^ Another process for hydrometallurgy is more advantageous based on time and energy consumption. Acid leaching can effectively extract valuable metals from black powder into the leachate within a short duration (30 min), as the metals are predominantly present in the form of oxides that readily react with acids.^41^ However, the acidic environment is also suitable for the leaching of impurities, necessitating a series of complex purification procedures during the subsequent treatments. Even worse, the regeneration of cathode materials, which involves a co-precipitation process, requires an alkaline environment, inherently conflicting with the acidic nature of the leachate. Conversely, alkaline leaching can overcome the challenges presented by acid leaching, yet it is predominantly hindered by a low reaction rate. Therefore, the strategy we proposed in this work aims to address the issue of a slow reaction rate, offering a promising pathway towards a sustainable, shortened, highly efficient closed-loop recycling system.

Although alkaline leaching has hardly garnered significant attention, the related literature studies have been reviewed with a focus on leaching time, temperature, ammonia concentration, and the most critical indicator, the leaching efficiency of valuable metals, as listed in Fig. 6b.^9–11,13–16,20,22,31,36,42–47^ A brief duration for a recycling process signifies a higher processing capacity per unit time. Benefiting from the galvanic cell interaction, the time necessary for the procedure in our work is significantly reduced to approximately 10 min, which is at the leading edge for all ammonia leaching methods. Moreover, the ultimate and most critical performance indicator is the leaching efficiency of the valuable metals. Comprehensively, the strategy proposed in this work achieves a well-balanced optimization in the aforementioned four aspects, exhibiting a superior leaching efficiency within a shortened time for recycling spent LIBs. Compared to the traditional solid-to-liquid reaction with the shrinking core model, the solid-to-solid reaction with galvanic cell interaction substantially addresses the inherent issue of sluggish leaching efficiency (Fig. 6b).

**Fig. 7:**
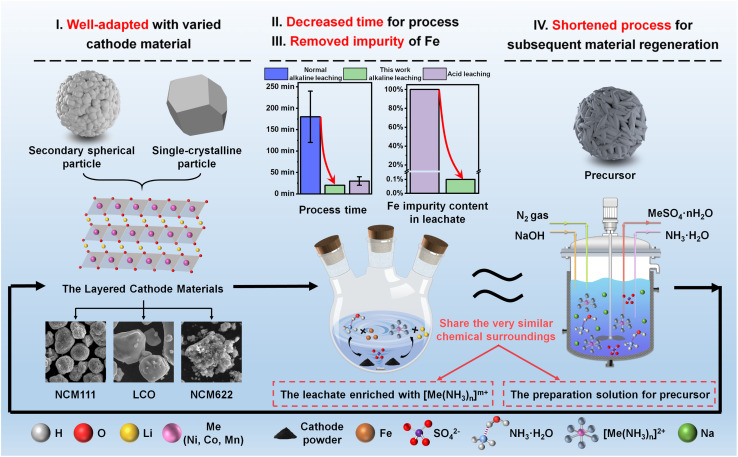
The advantages of alkaline leaching in the recycling of spent cathode materials.

Obviously, the alkaline-leaching process employed in this work offers four key advantages that address the challenges associated with establishing a sustainable and closed-loop recycling system, as illustrated in Fig. 7. The first advantage is well-adapted with various cathode materials. Normally, the diversity in particle size and element composition of spent cathodes necessitates a process with considerable adaptability. Empirical evidence presented in this work substantiates the robustness of the alkaline-leaching technique, demonstrating its efficacy in accommodating the recycling of various cathode materials, particularly for those featuring a layered crystallographic structure. Secondly, based on the proposed galvanic cell interaction, the rate of the redox reaction is significantly enhanced, culminating in a marked reduction in leaching time and a concomitant enhancement of metal extraction efficiency. Compared to the reported alkaline-leaching process, and even the acid-leaching process, our strategy also can exhibit high competitiveness. Thirdly, the laborious process of impurity removal may be substantially alleviated, as the majority of metallic impurities are sequestered in the form of hydroxides, facilitating their precipitation and subsequent separation. Especially Fe, which is usually observed in spent LIBs, can be completely removed under an alkaline atmosphere. Last but not least, material regeneration is pivotal to achieving the objective of a closed-loop recycling system. This leachate derived from the alkaline-leaching process exhibits an analogous chemical environment to an ammonia complex system, which can be directly utilized for preparing a cathode precursor during the co-precipitation process, thereby obviating the need for extensive compositional balance between acidity and alkalinity. Moreover, Li^+^ can be precipitated and recollected after the co-precipitation process for the subsequent calcination process, as exhibited in Fig. S21a.† Therefore, the regenerated material NCM523 has been successfully fabricated and demonstrated a commendable electrochemical performance, achieving an excellent capacity retention of 94.5% after 180 charge/discharge cycles, as displayed in Fig. S21b,† which totally satisfies the requirement of commercial cathode materials.

## Conclusions

An enhanced alkaline-leaching strategy with ultrafast efficiency is proposed in this work, which is expected to be a pivotal component in the establishment of a sustainable and closed-loop recycling for spent LIBs. The incorporation of a galvanic cell interaction substantially addresses the inherent issue of suboptimal leaching efficiency, manifesting a marked increase in the leaching efficiency. Particularly, 99.0% of Li, 93.0% of Ni, and 82.5% of Co can be extracted within a shortened time of 10 min. Compared to the conventional solid-to-liquid leaching process with a shrinking core model, the self-built solid-to-solid reaction with galvanic cell interaction exhibits a unique reaction mechanism. Based on the ultra-fast electron transfer, it supplants the interface reaction controlled by a slower diffusion process, leading to obvious improvements in reducing the leaching time and augmenting the overall leaching efficiency simultaneously. Moreover, the alkaline chemical environment not only ensures that most of the metallic impurities are precipitated into the leaching residue, but also contributes in a direct utilization of the ammonia-based leachate for cathode precursor regeneration. More importantly, the adoptability of the galvanic-cell-assisted alkaline-leaching process is further confirmed through its capacity to accommodate a variety of spent cathode materials. The deployment of the alkaline-leaching process is anticipated to facilitate the recycling process of spent LIBs, thereby enhancing the feasibility of implementing a sustainable closed-loop recycling system.

## Data availability

The data that support the findings of this study are available in the main text and the ESI.†

## Author contributions

Long Ye and Xing Ou conceived and designed the project. Long Ye, Zhilong Xu, Haiqiang Gong, Zhiming Xiao performed the analysis and prepared the manuscript. Bao Zhang, Lei Ming and Xing Ou coordinated and supervised the project. All authors contributed to the writing and editing of the manuscript.

## Conflicts of interest

There are no conflicts to declare.

## Supplementary Material

SC-OLF-D4SC06076H-s001
