# Supplementary material for: An ultra-fast reaction process for recycling lithium ion batteries via galvanic cell interaction
Source: Chem Sci. 2024 Nov 18;16(1):297–309. doi: 10.1039/d4sc06076h (PMC11600130; doi:10.1039/d4sc06076h)
Supplement: SC-016-D4SC06076H-s001 [file SC-016-D4SC06076H-s001.pdf]

Supplementary Information (SI) for Chemical Science.
This journal is © The Royal Society of Chemistry 2024

# Supporting Information

## An Ultra-fast Reaction Process for Recycling Lithium Ion Batteries *via* Galvanic Cell Interaction

*National Engineering Laboratory for High-Efficiency Recovery of Refractory Nonferrous Metals, School of Metallurgy and Environment, Central South University, Changsha 410083, PR China*

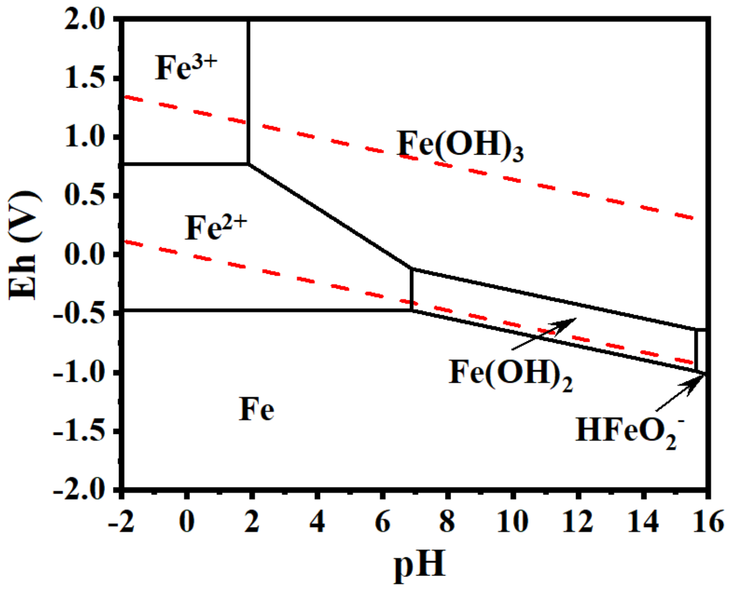


**Figure S1.** The Porbaix diagram of Fe-$H_2O$.

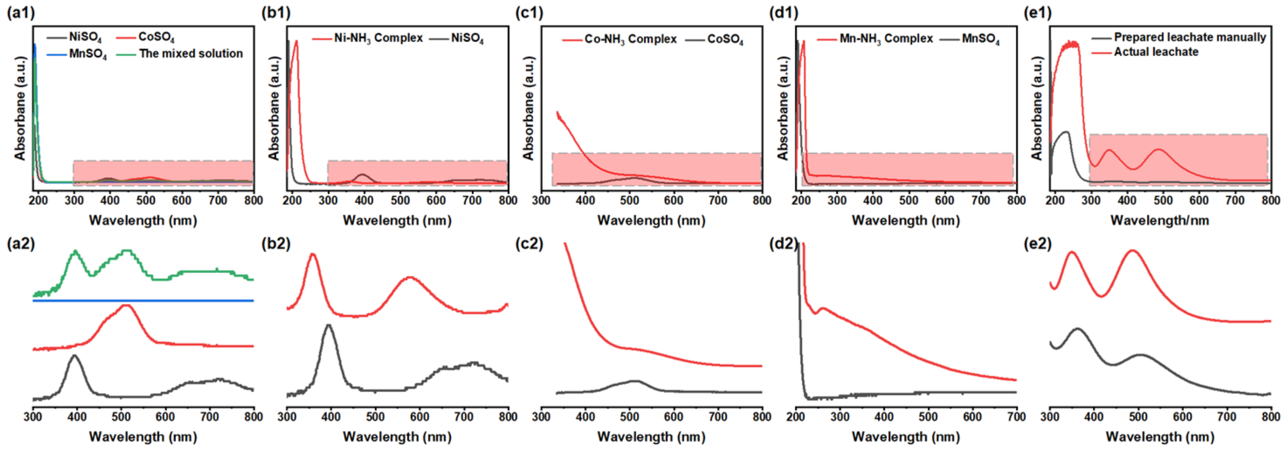


**Figure S2.** The UV-vis results of (a1-d1 and a2-d2) $Ni^{2+}$, $Co^{2+}$, $Mn^{2+}$ in two medias of water and leaching reagent, (e1 and e2) the actual leachate and prepared leachate manually.

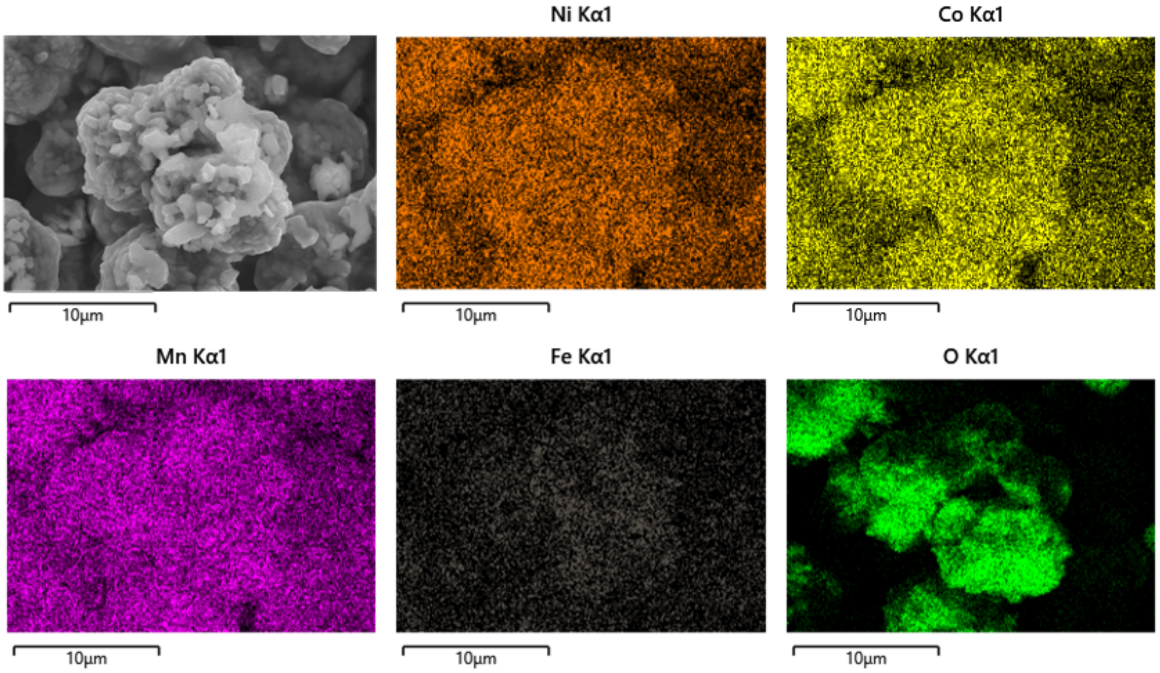


**Figure S3.** The EDS iamges for the NCM111 electrode after the external circuit detection test.

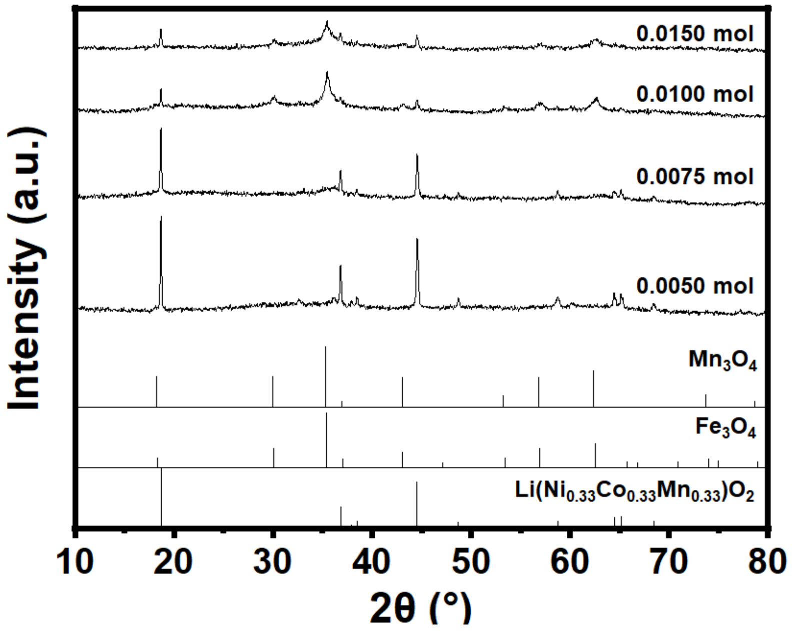


**Figure S4.** The XRD patterns of the leaching residues vary with a gradient of $FeSO_4$ usage.

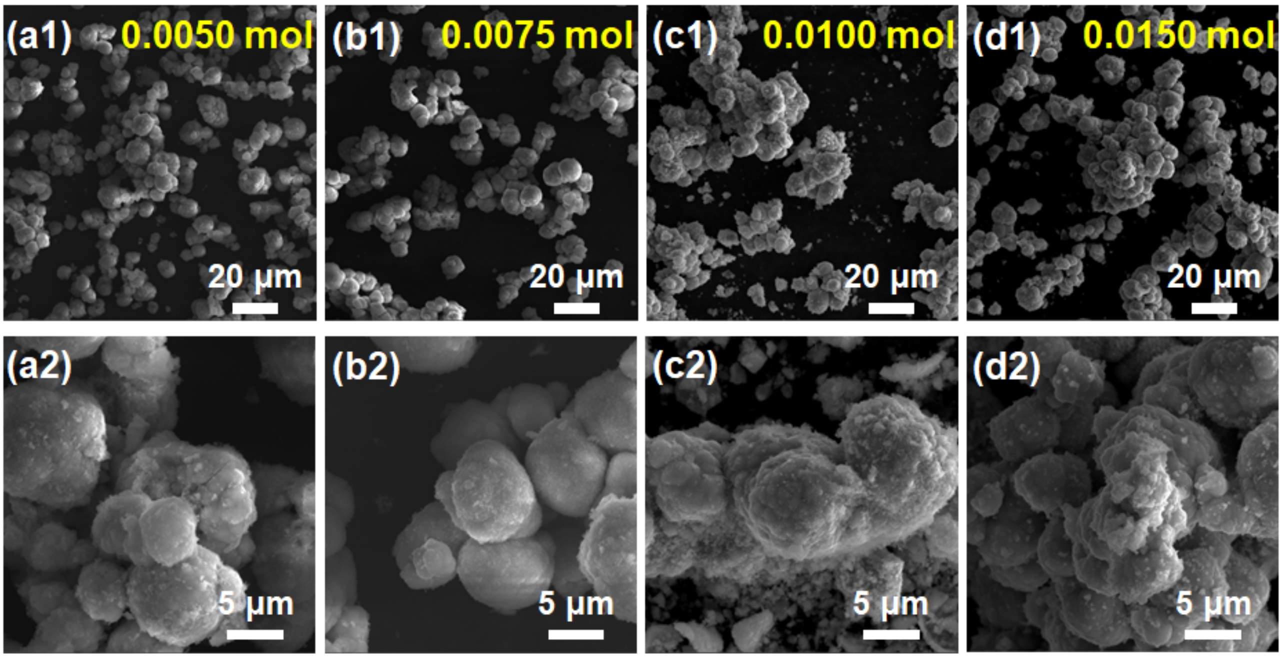


**Figure S5.** The SEM images of the leaching residues vary with a gradient of $FeSO_4$ usage.

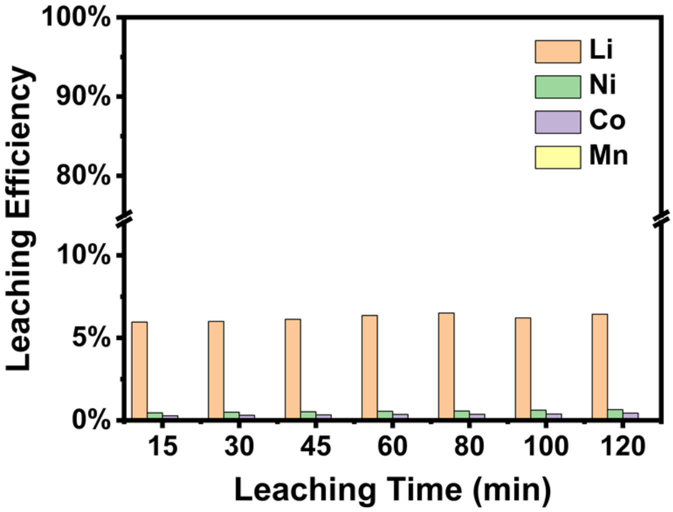


**Figure S6**. The leaching results without adding $FeSO_4$.

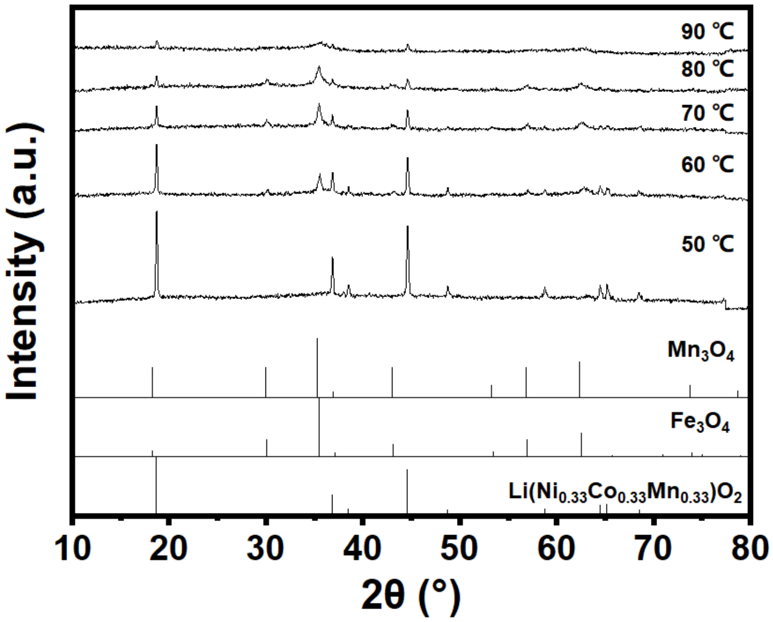


**Figure S7.** The XRD patterns of the leaching residues vary with a temperature gradient.

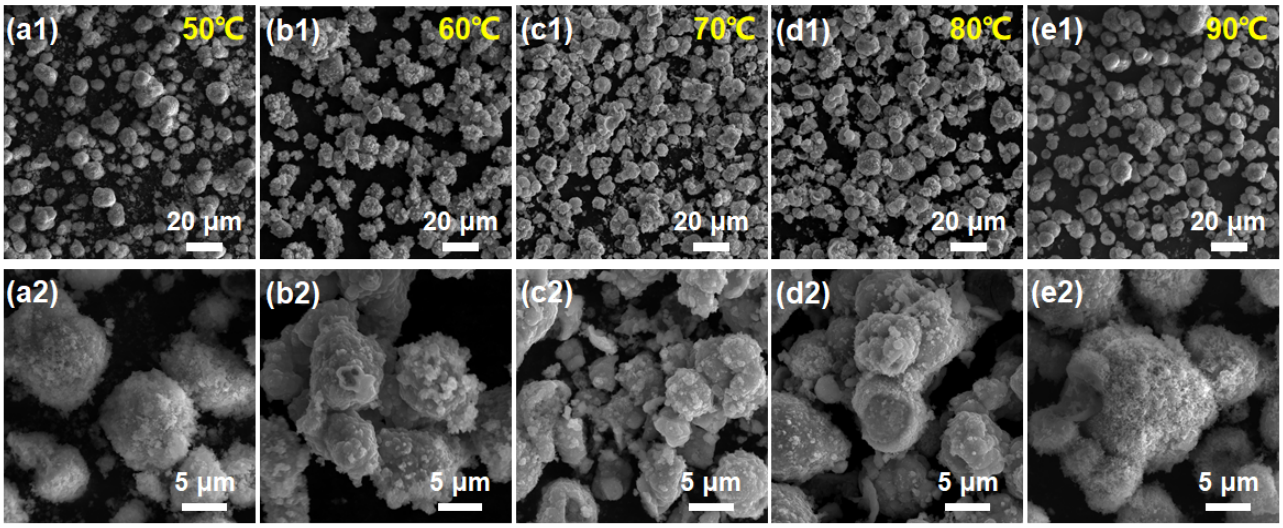


**Figure S8.** The SEM images of the leaching residues with a temperature gradient.

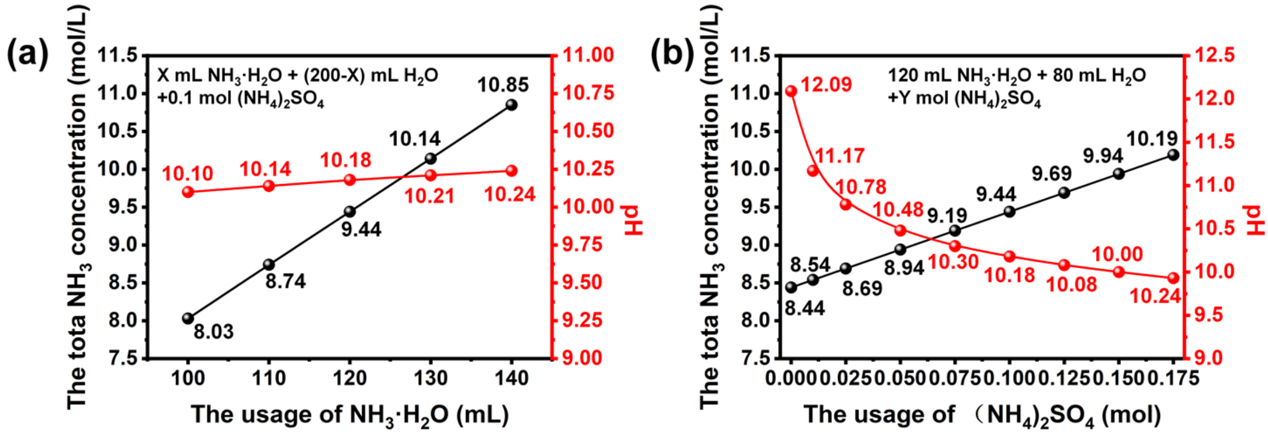


(c)

| Agents | The usage amount | | | | |
|---|---|---|---|---|---|
| $NH_3 \cdot H_2O$ (X mL) | 30 | 45 | 60 | 80 | 120 |
| $H_2O$ (200-X mL) | 170 | 155 | 140 | 120 | 80 |
| $(NH_4)_2SO_4$ (Y mol) | 0.025 | 0.038 | 0.05 | 0.067 | 0.1 |
| Total $NH_3$ (mol/L) | 2.36 | 3.54 | 4.72 | 6.29 | 9.44 |
| pH | 10.18 | 10.18 | 10.18 | 10.18 | 10.18 |

**Figure S9.** The changes of pH and total $NH_3$ concentration with elevated usage of (a) $NH_3 \cdot H_2O$ and (b) $(NH_4)_2SO_4$. (c) The changes as adjusting $NH_3 \cdot H_2O$ and $(NH_4)_2SO_4$ in euqal proportion.

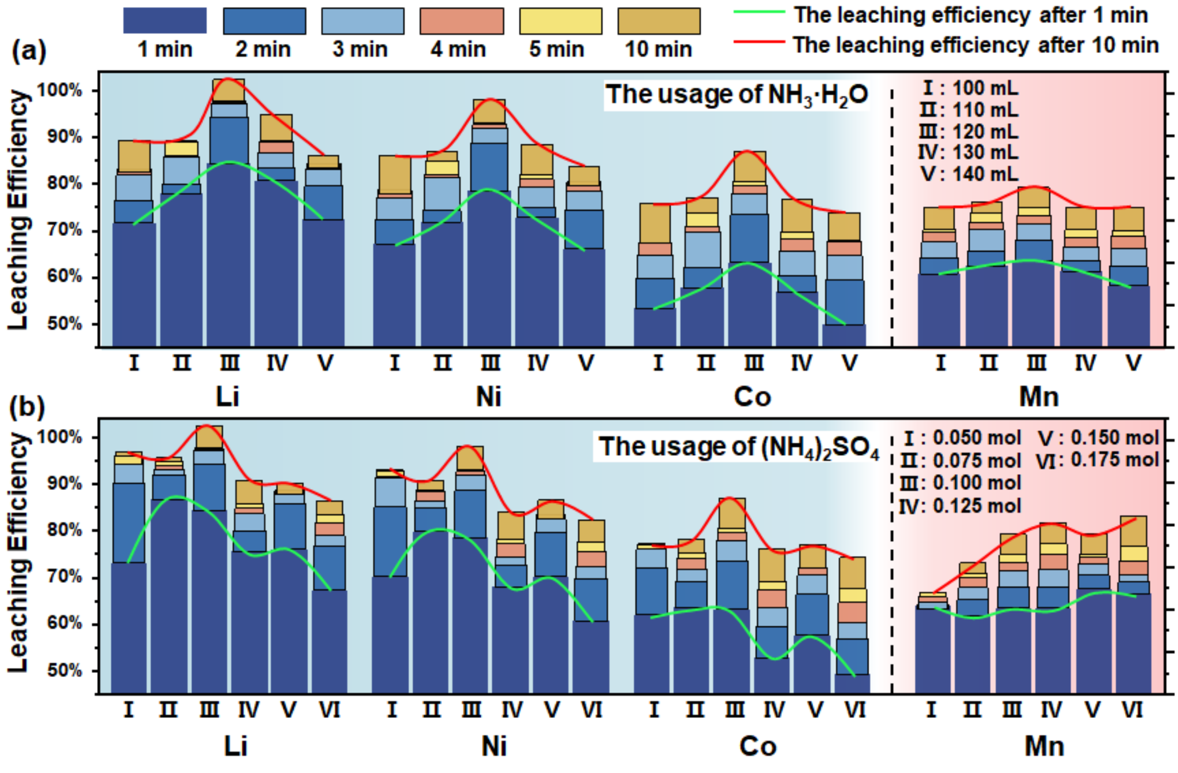


**Figure S10**. The leaching efficiency of valuable metals changes with the gradient of (a) $NH_3 \cdot H_2O$ usage, and (b) $(NH_4)_2SO_4$ usage.

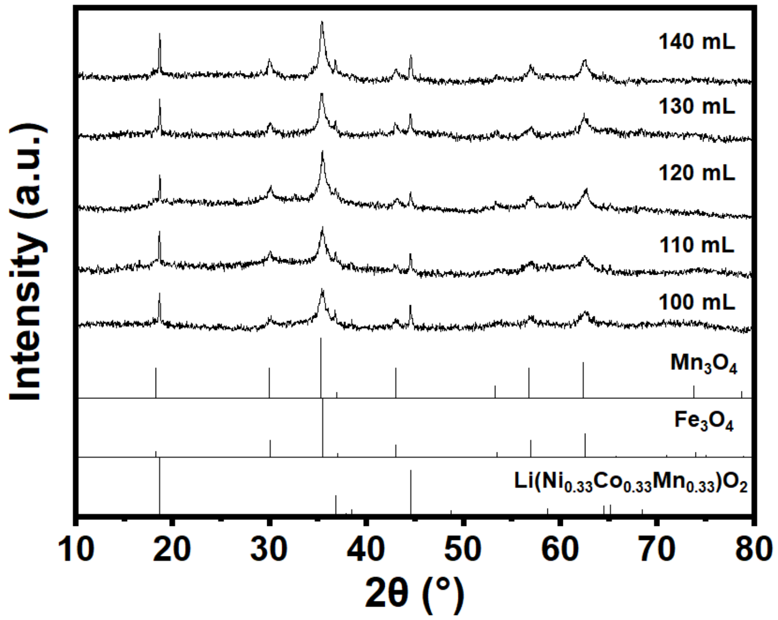


**Figure S11.** The XRD patterns of the leaching residues vary with a gradient of $NH_3{\cdot}H_2O$ usage.

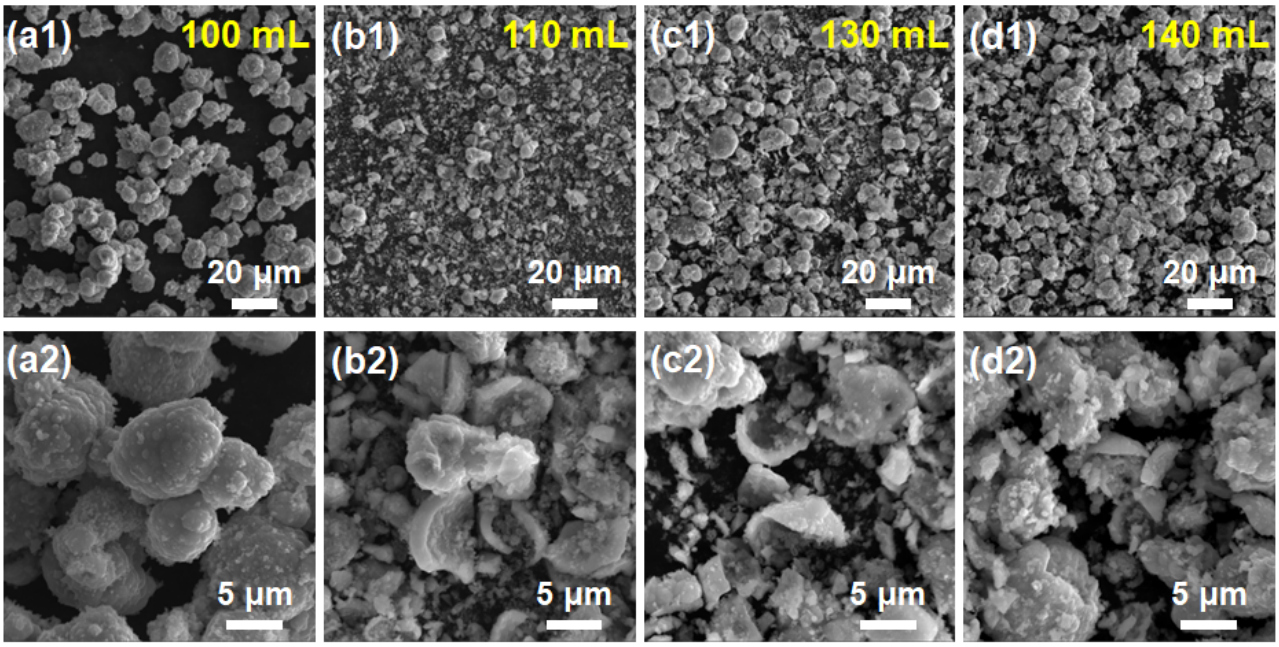


**Figure S12.** The SEM images of the leaching residues vary with a gradient of $NH_3{\cdot}H_2O$ usage.

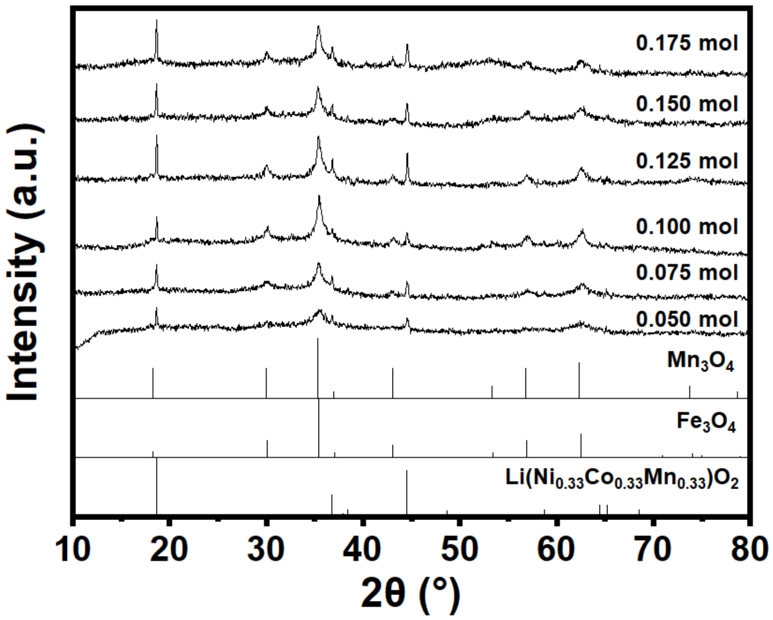


**Figure S13.** The XRD patterns of the leaching residues vary with a gradient of $(NH_4)_2SO_4$ usage.

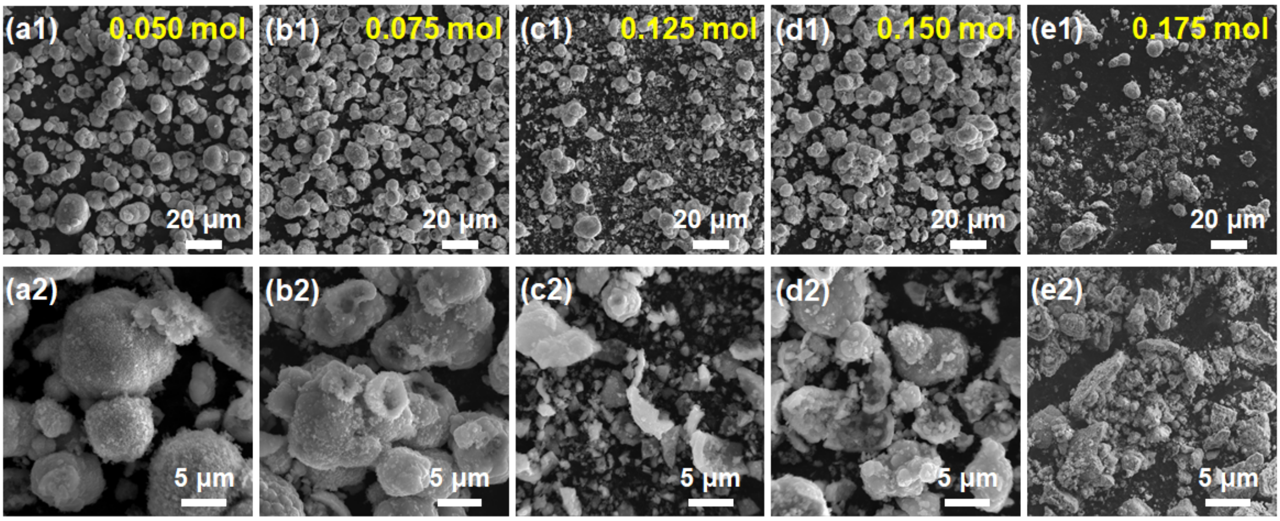


**Figure S14.** The SEM images of the leaching residues vary with a gradient of $(NH_4)_2SO_4$ usage.

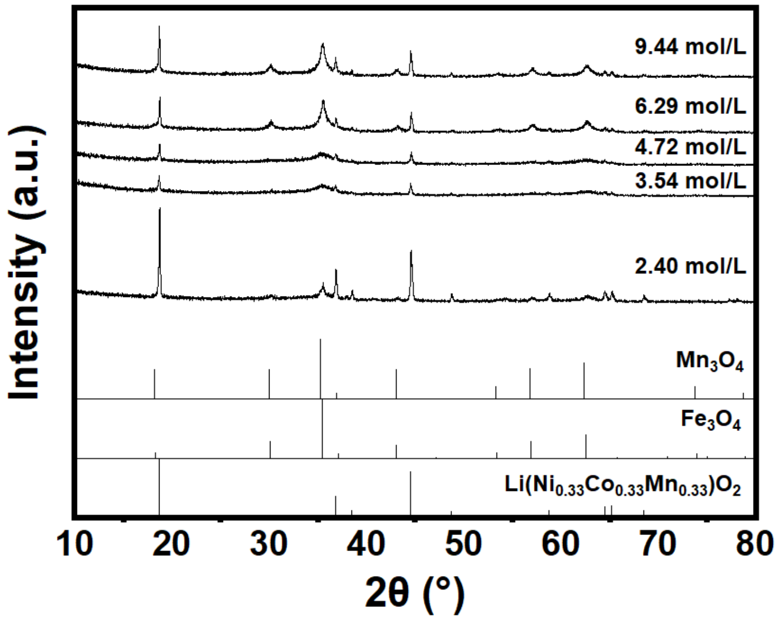


**Figure S15.** The XRD patterns of the leaching residues vary with a gradient in the concentration of $NH_3$.

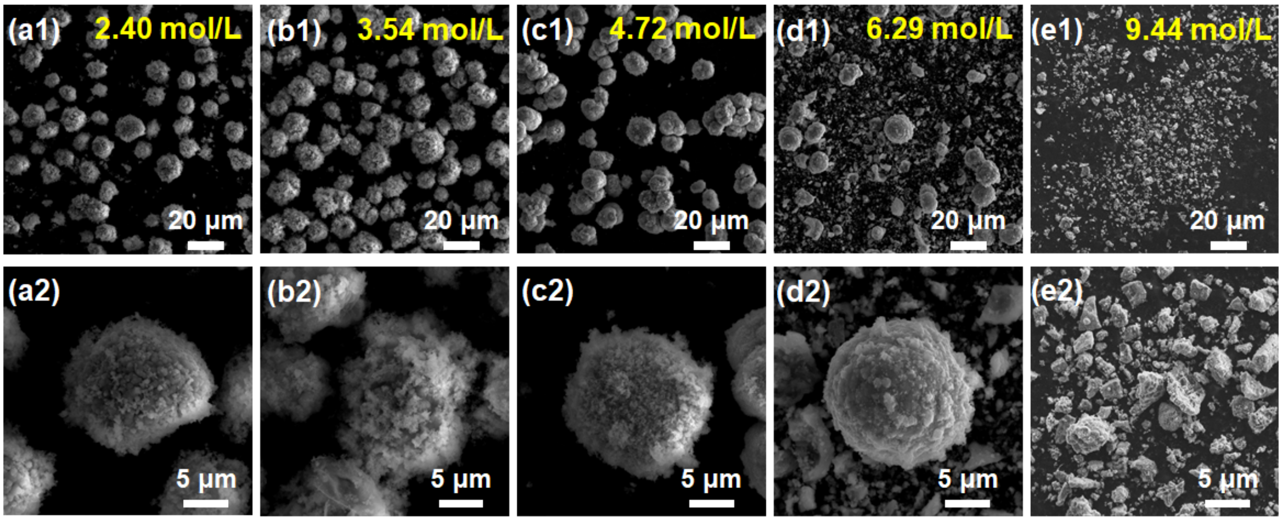


**Figure S16.** The SEM images of the leaching residues vary with a gradient in the concentration of $NH_3$.

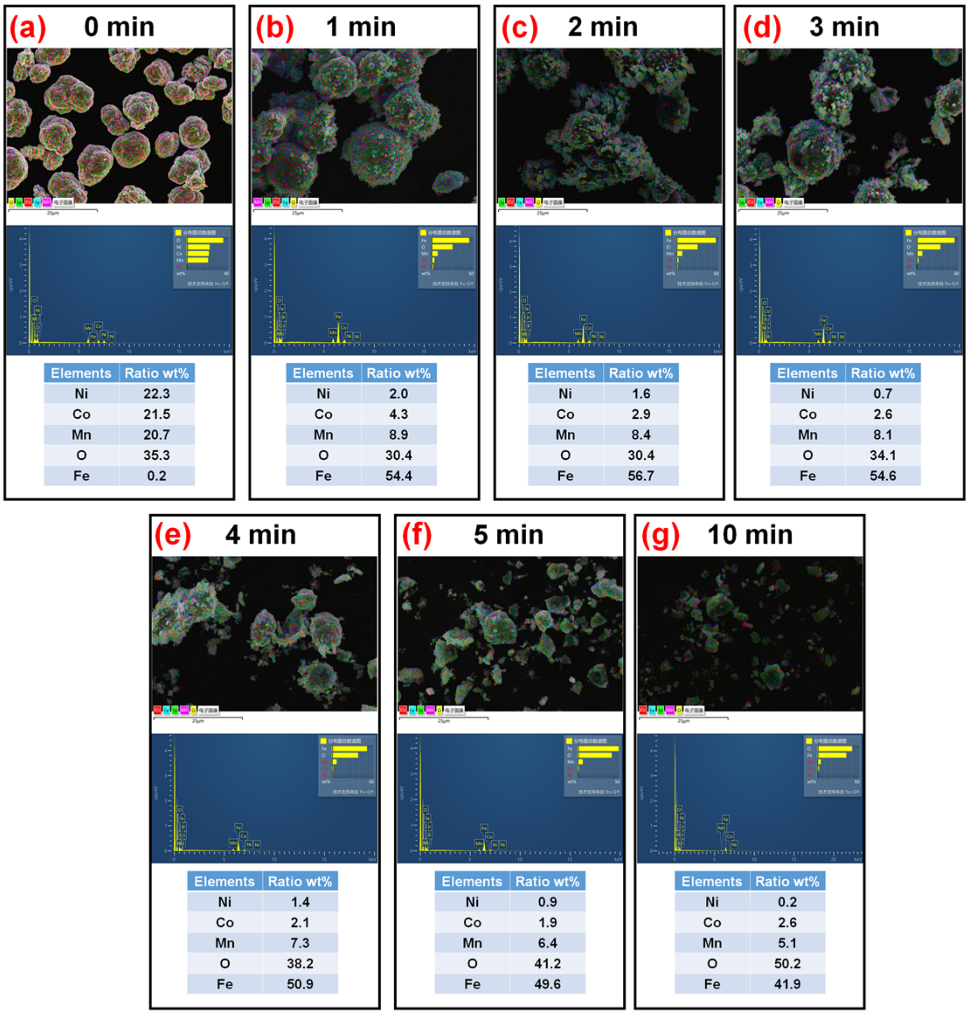


**Figure S17.** The EDS tests for the leaching residues at (a) 0 min, (b) 1 min, (c) 2 min, (d) 3 min, (e) 4 min, (f) 5 min, (g) 10 min.

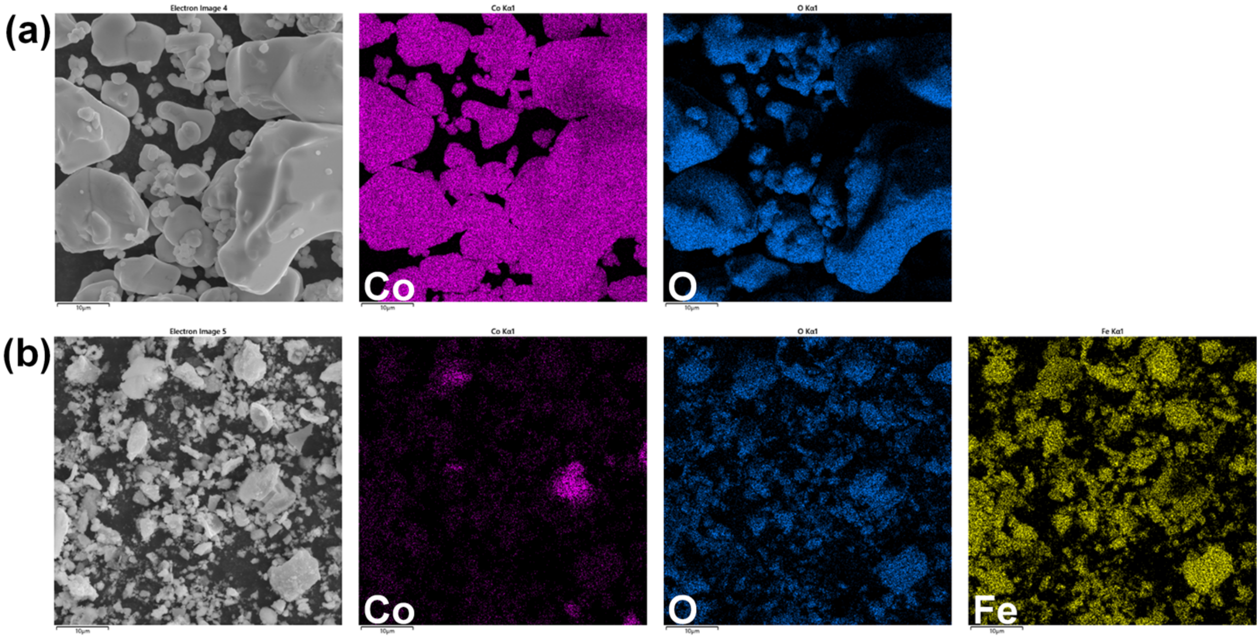


**Figure S18.** The EDS test of the LCO (a) before and (b) after leaching

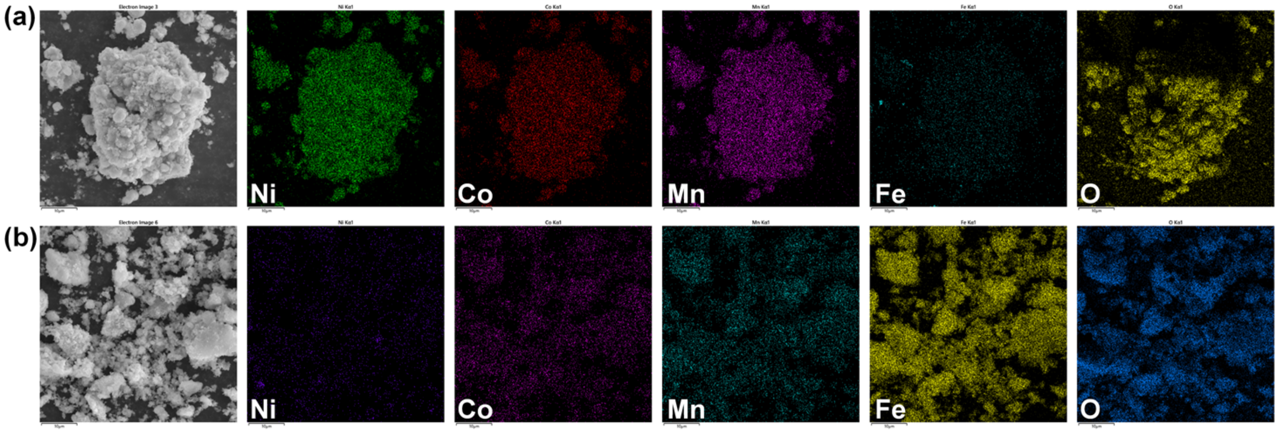


**Figure S19.** The EDS test of the NCM622 (a) before and (b) after leaching

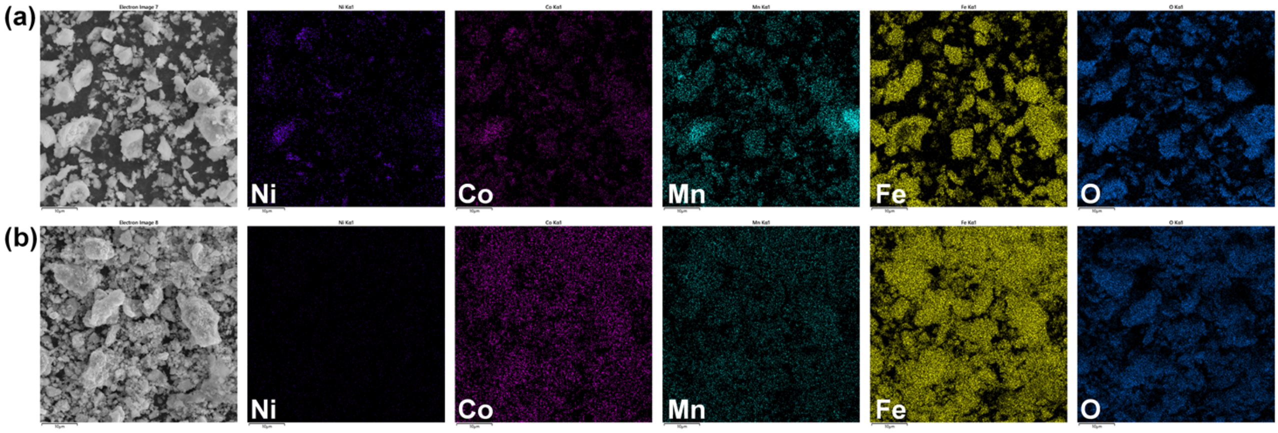


**Figure S20.** The EDS test of the two steps leaching: the (a) first and (b) second leaching residues

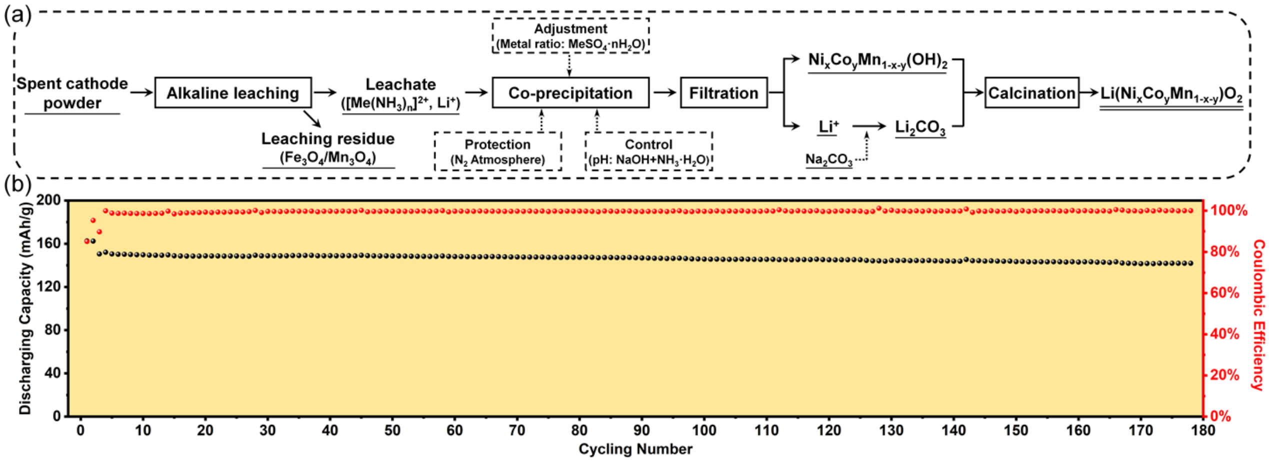


**Figure S21.** (a) The regeneration process. (b)The long-term cycling performance of regenerated NCM523 material

**Table S1.** The related information for Ni-$H_2O$ Pourbaix diagram

| Number | Chemical Equation | The calculated line when T = 298.15K |
|---|---|---|
| a | $2H^+ + 2e^- = H_2$ | E = -0.059*pH |
| b | $O_2 + 4H^+ + 4e^- = 2H_2O$ | E = 1.229 - 0.059*pH |
| 1 | $Ni^{3+} + e^- = Ni^{2+}$ | E = 1.840 |
| 2 | $Ni^{2+} + 2e^- = Ni$ | E = -0.236 |
| 3 | $Ni_2O_3 + 6H^+ = 2Ni^{3+} + 3H_2O$ | pH = -0.646 |
| 4 | $Ni(OH)_2 + 2H^+ = Ni^{2+} + 2H_2O$ | pH = 6.357 |
| 5 | $Ni_3O_4 + 2H_2O + 2H^+ + 2e^- = 3Ni(OH)_2$ | E = 0.807 - 0.059*pH |
| 6 | $Ni_3O_4 + 8H^+ + 2e^- = 3Ni^{2+} + 4H_2O$ | E = 1.935 - 0.237*pH |
| 7 | $Ni(OH)_2 + 2H^+ + 2e^- = Ni + 2H_2O$ | E = 0.140 - 0.059*pH |
| 8 | $Ni_2O_3 + 6H^+ + 2e^- = 2Ni^{2+} + 3H_2O$ | E = 1.725 - 0.178*pH |
| 9 | $3Ni_2O_3 + 2H^+ + 2e^- = 2Ni_3O_4 + H_2O$ | E = 1.305 - 0.059*pH |

**Table S2.** The related information for Co-$H_2O$ Pourbaix diagram

| Number | Chemical Equation | The calculated line when T = 298.15K |
|---|---|---|
| a | $2H^+ + 2e^- = H_2$ | E = -0.059*pH |
| b | $O_2 + 4H^+ + 4e^- = 2H_2O$ | E = 1.229 - 0.059*pH |
| 1 | $Co^{3+} + e^- = Co^{2+}$ | E = 1.953 |
| 2 | $Co^{2+} + 2e^- = Co$ | E = -0.282 |
| 3 | $Co_2O_3 + 6H^+ = 2Co^{3+} + 3H_2O$ | pH = -3.348 |
| 4 | $Co(OH)_2 + 2H^+ = Co2^+ + 2H_2O$ | pH = 6.530 |
| 5 | $Co_3O_4 + 2H_2O + 2H^+ + 2e^- = 3Co(OH)_2$ | E = 0.485 - 0.059*pH |
| 6 | $Co_3O_4 + 8H^+ + 2e^- = 3Co^{2+} + 4H_2O$ | E = 1.644 - 0.237*pH |
| 7 | $Co(OH)_2 + 2H^+ + 2e- = Co + 2H_2O$ | E = 0.104 - 0.059*pH |
| 8 | $Co_2O_3 + 6H^+ + 2e^- = 2Co^{2+} + 3H_2O$ | E = 1.358 - 0.178*pH |
| 9 | $3Co_2O_3 + 2H^+ + 2e^- = 2Co_3O_4 + H_2O$ | E = 0.786 - 0.059*pH |

**Table S3.** The related information for Mn-$H_2O$ Pourbaix diagram

| Number | Chemical Equation | The calculated line when T = 298.15K |
|---|---|---|
| a | $2H^+ + 2e^- = H_2$ | E = -0.059*pH |
| b | $O_2 + 4H^+ + 4e^- = 2H_2O$ | E = 1.229 - 0.059*pH |
| 1 | $Mn^{3+} + e^- = Mn^{2+}$ | E = 1.542 |
| 2 | $Mn^{2+} + 2e^- = Mn$ | E = -1.182 |
| 3 | $MnO_2 + 4H^+ + 2e^- = 2Mn^{2+} + 3H_2O$ | E = 1.229 - 0.118*pH |
| 4 | $Mn(OH)_2 + 2H^+ = Mn^{2+} + 2H_2O$ | pH = 7.282 |
| 5 | $Mn_3O_4 + 2H_2O + 2H^+ + 2e^- = 3Mn(OH)_2$ | E = 0.519- 0.059*pH |
| 6 | $Mn_3O_4 + 8H^+ + 2e^- = 3Mn^{2+} + 4H_2O$ | E = 1.812 - 0.237*pH |
| 7 | $Mn(OH)_2 + 2H^+ + 2e^- = Mn + 2H_2O$ | E = -0.751 - 0.059*pH |
| 8 | $2MnO_2 + 2H^+ + 2e^- = Mn_2O_3 + H_2O$ | E = 0.974 - 0.059*pH |
| 9 | $Mn_2O_3 + 6H^+ + 2e^- = 2Mn^{2+} + 3H_2O$ | E = 1.484 - 0.178*pH |
| 10 | $3Mn_2O_3 + 2H^+ + 2e^- = 2Mn_3O_4 + H_2O$ | E = 0.829 - 0.059*pH |
| 11 | $MnO_4^- + e^- = MnO_4^{2-}$ | E = 0.554 |
| 12 | $MnO_4^- + 4H^+ + 3e^- = MnO_2 + 2H_2O$ | E = 1.700 - 0.079*pH |
| 13 | $MnO_4^{2-} + 2e^- + 4H^+ = MnO_2 + 2H_2O$ | E = 2.273 - 0.118*pH |

**Table S4.** The related information for Fe-$H_2O$ Pourbaix diagram

| Number | Chemical Equation | The calculated line when T = 298.15K |
|---|---|---|
| a | $2H^+ + 2e^- = H_2$ | E = -0.059*pH |
| b | $O_2 + 4H^+ + 4e^- = 2H_2O$ | E = 1.229 - 0.059*pH |
| 1 | $Co^{3+} + e^- = Co^{2+}$ | E = 1.953 |
| 2 | $Co^{2+} + 2e^- = Co$ | E = -0.282 |
| 3 | $Co_2O_3 + 6H^+ = 2Co^{3+} + 3H_2O$ | pH = -3.348 |
| 4 | $Co(OH)_2 + 2H^+ = Co^{2+} + 2H_2O$ | pH = 6.530 |
| 5 | $Co_3O_4 + 2H_2O + 2H^+ + 2e^- = 3Co(OH)_2$ | E = 0.485 - 0.059*pH |
| 6 | $Co_3O_4 + 8H^+ + 2e^- = 3Co^{2+} + 4H_2O$ | E = 1.644 - 0.237*pH |
| 7 | $Co(OH)_2 + 2H^+ + 2e^- = Co + 2H_2O$ | E = 0.104 - 0.059*pH |
| 8 | $Co_2O_3 + 6H^+ + 2e^- = 2Co^{2+} + 3H_2O$ | E = 1.358 - 0.178*pH |
| 9 | $3Co_2O_3 + 2H^+ + 2e^- = 2Co_3O_4 + H_2O$ | E = 0.786 - 0.059*pH |

**Table S5.** The leaching efficiency of five independent experiment

| Element | Time/min | Leaching efficiency | | | | | Average | SD |
|---|---|---|---|---|---|---|---|---|
| Li | 1 | 0.801 | 0.84304 | 0.89804 | 0.77954 | 0.75744 | 0.81581 | 0.05578 |
| | 2 | 0.85976 | 0.94167 | 0.95961 | 0.84909 | 0.83756 | 0.88954 | 0.05668 |
| | 3 | 0.93056 | 0.97233 | 0.9572 | 0.89394 | 0.90057 | 0.93092 | 0.03426 |
| | 4 | 0.89009 | 0.97323 | 0.95906 | 0.94032 | 0.93412 | 0.93936 | 0.03159 |
| | 5 | 0.96797 | 0.97804 | 0.971 | 0.96319 | 0.96214 | 0.96847 | 0.00645 |
| | 10 | 0.97359 | 1.02312 | 0.9951 | 0.99958 | 1.00822 | 0.99992 | 0.01819 |
| Ni | 1 | 0.73245 | 0.78575 | 0.81633 | 0.68059 | 0.6533 | 0.73368 | 0.06860 |
| | 2 | 0.79886 | 0.88537 | 0.84698 | 0.74807 | 0.73169 | 0.80219 | 0.06488 |
| | 3 | 0.87943 | 0.9191 | 0.87063 | 0.79156 | 0.79213 | 0.85057 | 0.05663 |
| | 4 | 0.84483 | 0.92625 | 0.85717 | 0.8404 | 0.82571 | 0.85887 | 0.03931 |
| | 5 | 0.92792 | 0.92919 | 0.88466 | 0.86443 | 0.86185 | 0.89361 | 0.03310 |
| | 10 | 0.94858 | 0.98121 | 0.90292 | 0.90988 | 0.9068 | 0.92988 | 0.03407 |
| Co | 1 | 0.56258 | 0.63224 | 0.65772 | 0.55206 | 0.50508 | 0.58194 | 0.06214 |
| | 2 | 0.64869 | 0.73422 | 0.6957 | 0.62715 | 0.58869 | 0.65889 | 0.05716 |
| | 3 | 0.74073 | 0.77922 | 0.73037 | 0.67874 | 0.66041 | 0.71789 | 0.04816 |
| | 4 | 0.72521 | 0.79604 | 0.72648 | 0.73242 | 0.70154 | 0.73634 | 0.03540 |
| | 5 | 0.80229 | 0.80633 | 0.75881 | 0.76033 | 0.74305 | 0.77416 | 0.02838 |
| | 10 | 0.83893 | 0.87046 | 0.79254 | 0.81937 | 0.80303 | 0.82487 | 0.03094 |
| Mn | 1 | 0.18081 | 0.20421 | 0.22817 | 0.22234 | 0.18993 | 0.20509 | 0.02031 |
| | 2 | 0.2501 | 0.25336 | 0.25247 | 0.28675 | 0.26182 | 0.2609 | 0.01511 |
| | 3 | 0.32075 | 0.2899 | 0.28386 | 0.34473 | 0.33325 | 0.31450 | 0.02669 |
| | 4 | 0.32991 | 0.31156 | 0.29437 | 0.39923 | 0.37753 | 0.34252 | 0.04438 |
| | 5 | 0.37395 | 0.32914 | 0.3151 | 0.4266 | 0.41807 | 0.37257 | 0.05045 |
| | 10 | 0.38024 | 0.37762 | 0.34605 | 0.49074 | 0.4812 | 0.41517 | 0.06610 |
| Fe | 1 | 0.07277 | 0.12247 | 0.11102 | 0.14909 | 0.17007 | 0.12508 | 0.03722 |
| | 2 | 0.01803 | 0.03946 | 0.03205 | 0.07709 | 0.08575 | 0.05048 | 0.02944 |
| | 3 | 8.5E-04 | 0.01665 | 0.01305 | 0.03871 | 0.03841 | 0.02154 | 0.01661 |
| | 4 | 6.3E-05 | 0.00433 | 0.00548 | 0.01379 | 0.01344 | 0.00742 | 0.00600 |
| | 5 | 8.2E-05 | 0.00269 | 0.00328 | 0.00501 | 0.00329 | 0.00287 | 0.00178 |
| | 10 | 0.00209 | -0.0019 | 0.00318 | 0.00144 | 0.0014 | 0.00125 | 0.00188 |
